# Rip current detection in nearshore areas through UAV video analysis using optical flow and almost local-isometric embedding techniques on the sphere

**DOI:** 10.1371/journal.pone.0360672

**Published:** 2026-10-29

**Authors:** Kaiqi Yang, Stephen B. Leatherman, Anchen Sun

**Affiliations:** 1 Department of Mathematics, University of Miami, Coral Gables, Florida, United States of America; 2 Department of Earth and Environment, Florida International University, Miami, Florida, United States of America; 3 Department of Electrical and Computer Engineering, University of Miami, Coral Gables, Florida, United States of America; Islamia University of Bahawalpur: The Islamia University of Bahawalpur Pakistan, PAKISTAN

## Abstract

Rip currents cause most surf-beach lifeguard rescues and most beach drowning incidents, yet automated detection relies on costly instrumentation that scales poorly across the many beaches that lack lifeguards. We propose an end-to-end pipeline producing pixel-level rip-current likelihood maps directly from raw unmanned aerial vehicle (UAV) video without manual ground-control points — to our knowledge the first to do so from uncalibrated drone video, prior automated approaches operating at video-clip granularity or requiring surveyed camera installations. The pipeline composes (i) dense optical-flow estimation with five classical algorithm variants; (ii) a sphere-aware projection mapping pixel coordinates to local-isometric ground coordinates, with a tilt-aware Jacobian correction of per-pixel velocity; (iii) a CNN six-class scene parser (sky/sea/land/foam/structure/void), trained on frames verified by dense pixel-matching to contain no extraction from either evaluation video; (iv) a per-pixel temporal accumulator flagging pixels whose flow direction agrees with the locally-estimated offshore direction within $\pm 90^{\circ }$ — the physical boundary of seaward-directed flow, shown robust in an 18-scene sensitivity sweep; and (v) post-processing by Gaussian smoothing (σ=25 px) and a normalized cross-algorithm ensemble. We evaluate on two UAV-captured rip-current videos at Palm Beach and Haulover Park, Florida, annotated by consensus among three coastal-science domain experts. At the physically-neutral $T=90^{\circ }$ default the framework attains precision-recall AUC 0.636 at Palm Beach and 0.660 at Haulover; per-scene selection — of the angular tolerance, ensemble membership, mixing weight, sphere tilt, and which modules are engaged — against the same expert annotation used for evaluation raises these to 0.7066 and 0.7040, reported as an optimistic upper bound rather than an expected-performance estimate. We release the source code, the trained sea-mask refiner, a YOLOv8m-seg deep-learning baseline, and the evaluation scripts. Evaluation is limited to these two expert-annotated scenes, and the method requires visible surface tracers (foam, dye, or sediment fronts) advected by the current.

## Introduction

Rip currents are responsible for the majority of surf-beach lifeguard rescues and most beach drowning incidents [1], yet most U.S. beaches lack lifeguards because professional staffing is costly [2]. A low-cost, automated detection system that can be deployed at unguarded beaches would directly reduce drowning risk. This paper proposes such a system based on Unmanned Aerial Vehicle (UAV) video, and evaluates its effectiveness against expert-annotated ground truth.

Existing rip-current measurement techniques are accurate but operationally heavy. Analytic models built on the linear stability of time-averaged flow [3] require capacitance wave gauges and acoustic Doppler velocimeters (ADVs) deployed in energetic surf-zone conditions, demanding trained operators and exposing equipment to risk. Marine radars detect current velocity from backscatter off the rough ocean surface [4]. Numerical models such as ROMS-SWAN [5], Delft 3D [6], and FUNWAVE [7] predict rip currents from bathymetric and offshore boundary data, but require substantial pre-processing and are unsuitable for real-time monitoring. Video-based methods avoid most of this overhead [8]: prior work has used one-dimensional measures [9], two-dimensional cross-correlation [10], drifter tracking [11], and dense optical flow on UAV imagery [12]. We discuss these methods in detail in the next section and compare their effectiveness.

Recent advances in machine learning and multimedia analysis have made dense, real-time analysis of unstructured video data tractable [13]. Combined with the operational ease of UAVs, this opens a path to a video-only rip-current detection system. Compared to model-based approaches, UAV-video-based detection is (i) adaptable across locations and oceanographic conditions, (ii) low-cost in equipment and training, and (iii) responsive in near-real-time without bathymetric pre-processing. UAV deployment also has limitations that we acknowledge: weather sensitivity (winds ≥15 m/s typically ground a small UAV), battery-limited flight time on the order of 25 min, camera-motion artifacts when stabilization is imperfect, and regulatory constraints in many jurisdictions (e.g., FAA Part 107 in the U.S., or local airspace restrictions). These limitations bound the operating envelope of any UAV-based system, including ours.

Within the dense optical-flow line of work, the wavelet-based “Typhoon” algorithm produced the first quantitatively validated estimates of instantaneous surface currents from shore-based and UAV video, with bias verified against ADV measurements [12]. This and related work demonstrate that optical flow can recover surface velocity at the pixel level. We are not aware, however, of any prior end-to-end pipeline that takes raw UAV video as input, requires no manual ground-control points, and produces a pixel-level rip-current likelihood map as output. Prior automated work either operates at coarse video-clip granularity (e.g., [14]) or requires tower-mounted cameras with surveyed reference points (e.g., [15]); the gap our pipeline addresses is dense per-pixel rip-current scoring directly from drone video.

The primary objective of this study is to develop and validate an end-to-end pipeline that produces pixel-level rip-current likelihood maps directly from raw UAV video without manual ground-control points; secondary objectives are to quantify the pipeline’s sensitivity to its angular-tolerance parameter across scenes and to bound the error of planar versus sphere-aware geometric projection over operating altitude. Our specific contributions are three core methodological components, together forming an end-to-end pipeline that produces pixel-level rip-current likelihood maps from raw UAV video with no manual ground-control points required at deployment time:

1. **Ground-control-point-free geometric normalization.** A sphere-aware projection (“almost local-isometric embedding on the sphere”) maps image-plane coordinates to local-isometric ground coordinates and applies a tilt-aware Jacobian to per-pixel velocity. The formulation is valid across imaging scales: planar homography is its small-footprint limit (which suffices at our 50–70 m operating altitude, as we quantify), while the same expression remains correct at higher altitudes and longer ranges where Earth curvature becomes non-negligible.
2. **Direction-consistent temporal accumulation.** A per-pixel accumulator separates the steady seaward drift that defines a rip from oscillatory wave motion by counting the frames in which the locally-estimated optical-flow direction lies within the offshore half-plane (within ±T of the locally-estimated offshore direction, with the physically-neutral default $T=90^{\circ }$). Because the zero-mean wave component averages out over the accumulation window while the persistent rip drift accumulates, the step performs coherent temporal integration of steady offshore motion; a direction-test-removed control in the Results quantifies how much of the final score this test contributes once segmentation gating and smoothing are engaged (+ 0.018 at the neutral default on Palm Beach, + 0.088 at the permissive per-scene setting).
3. **Modular learned-plus-deterministic post-processing.** A six-class CNN scene parser (U-Net, ResNet-18 encoder, 14.3 M parameters) supplies a per-frame sea probability P(sea∣frame) that gates the accumulator; a fixed Gaussian smoothing (σ=25 px) and a max-normalized cross-algorithm ensemble of HS- and LK-based variants then denoise and combine the heatmaps. With this block, the full pipeline attains precision-recall AUC 0.636 on Palm Beach and 0.660 on Haulover at the neutral $T=90^{\circ }$ default, rising to 0.7066 and 0.7040 under per-scene selection against the evaluation annotation (reported throughout as an upper bound, not an expected-performance estimate).

We validate these components with an angular-tolerance sensitivity sweep across 18 scenes, a planar-versus-sphere projection ablation over altitude, a leakage-controlled segmenter re-training, a short-window cross-scene test, and a YOLOv8m-seg deep-learning baseline (fine-tuned on the NTIRE 2026 RipDetSeg corpus of 29,307 rip-current images), and we release the source-code distribution, the trained weights, and the evaluation scripts.

The structure of this paper is organized as follows. The Related Work section reviews model-based and video-based rip-current detection. The Proposed Rip Current Detection Framework section details the pipeline: scene segmentation, dense optical-flow estimation, sphere-aware projection, offshore-direction estimation, and temporal accumulation together with its wave-noise error model. The Experiment Results section reports the evaluation on the Palm Beach and Haulover UAV scenes, including the multi-scene angular-tolerance sensitivity analysis, the empirical accumulation-length study, the CNN sea-mask refinement with its leakage verification, the spatial-smoothing and cross-algorithm ensemble step, the sphere-projection ablation and altitude bound, a short-window evaluation on an additional UAV clip under measurable camera motion, and the deep-learning baseline trained on the NTIRE 2026 RipDetSeg challenge dataset. The Conclusion and Further Work section discusses limitations, scope, and future work.

## Related work

### Model-based rip current detection methods

A variety of methodologies have been used by researchers to identify rip currents, with the most common approach being direct observation with the naked eye. There are several tell-tale signs of rip currents, including a tongue of sediment-laden water moving offshore, seaward movement of floating objects, unusual wave choppiness, foamy water in the outer edges of the rip head, gap in the breaking waves, and darker water that indicates the presence of a rip channel.

One study, referenced as [16], used water-filled polyethylene balls that were adjusted to be neutrally buoyant to detect rip currents. Comparisons with dye releases indicated that the movement of the freely-drifting balls represented the mean flow. Additionally, nearly-filled plastic jugs of water have been used to locate rips and provide an indication of Lagrangian trajectories, which is a low-cost method that is still widely used in developing countries [17]. This method can also provide an estimate of rip speed.

In recent years, more sophisticated instrumentation has been deployed at rip-prone beaches, such as tripod-mounted current meters and pressure sensors. However, there are challenges associated with this Eulerian method of rip measurement, including concerns about instrumentation placement and whether the current meter remains in the mean flow area of the rip. Additionally, logistical problems and safety constraints exist when installing instrumentation in a dangerous location [18].

In Australia, experiments involving individuals floating freely in rip currents have been conducted using theodolites [19] and more recently GPS technology. Lagrangian methods have gained popularity for measuring rip currents, with GPS-equipped drogues offering advantages such as shorter set-up time, safer deployment, increased mobility, and the ability to track the rip current path. MacMahan et al. extensively used GPS-controlled drogues to measure rip current velocity in California, France, and Australia, with real-time differential GPS drifters having an accuracy of less than 1 meter after carrier phase post-processing [20]. Non-differential GPS units, which are significantly less expensive, are now being used with great success to study rip currents [21].

Smith and Largier employed Doppler sonar to measure rip currents at the Scripps Institute of Oceanography pier in California [22]. While acoustic meters provide accurate measurements [9], they are expensive, unwieldy, and require significant set-up time, making them unsuitable for rapid deployments or long-term monitoring. Lushine developed the first rip forecast models [23], which were later refined by Lascody [24]. Lushine’s model quantified the rip threat by analyzing drowning, meteorological, and oceanographic records, and established the relationship between rip occurrence and wind and low tide. Lascody modified the model by incorporating swell wave data [24].

Numerical models that predict rip currents require detailed bathymetric and offshore boundary condition data. Several coupled 3-D circulation and wave propagation models have been developed to predict rip currents. Kumar et al. [5] coupled the circulation model ROMS with the wave propagation model SWAN to model rip currents. ROMS is a 3-D numerical bathymetry-following circulation model that solves finite difference approximations of Reynolds-averaged Navier-Stokes equations using hydrostatic and Boussinesq approximations with a split-explicit time stepping algorithm [5]. Brown et al. [6] applied the Delft 3-D circulation model to the nearshore with promising results. Wang et al. [7] used the FUNWAVE model, which utilizes the Boussinesq equations, to simulate rip currents off arc-shaped coastlines. These numerical models require significant amounts of initial data and processing time and are not suitable for real-time measurements of rip currents.

Video cameras have been a popular tool for measuring waves and currents at beaches for many years [16]. These cameras are typically mounted on buildings, poles, or balloons and use telephoto lenses to capture footage of the surf zone. Holman and Stanley used an Argus camera system at the Corps of Engineers Field Research Facility in Duck, North Carolina to obtain time-lapsed photography [25]. Google Earth and Bing satellite images can also be used to identify rip currents based on differential water coloration or sediment plumes. Optical methods, such as Particle Image Velocimetry (PIV), have been used to study rip currents [9,10]. These techniques use video footage to track the drift of sea foam in the surf zone and derive current velocity measurements. However, they require a set of visible ground control points with known locations and are limited by the location and placement of the camera, as well as the contrast of features in the images. Chickadel et al. developed an optical current meter algorithm that Fourier transforms space-time data into a frequency-wave number spectrum and then to a velocity spectrum [9].

Table 1 summarizes the model-based and instrumentation-heavy approaches discussed above, highlighting hardware cost, real-time capability, and the dependence on bathymetric or boundary data that limits scaling to many beaches. The video-based approaches we adopt avoid most of these constraints, at the cost of dependence on visible surface tracers (foam, sediment) and adequate lighting.

**Table 1:** Comparison of model-based rip-current detection methods. Method references: wave gauges + ADV [3], marine radar [4], numerical models [5–7], GPS drifters [20,21], video PIV [9,10].

Method	Cost	Real-time?	Scalability	Bathymetry?
Wave gauges + ADV	high	after deploy	one rip site	no
Marine radar	very high	yes	1–5 km range	no
Numerical models	low	offline	regional	yes
GPS drifters	moderate	yes	limited	no
Tower / pier PIV	moderate	yes	fixed site	no
**UAV video (this paper)**	low	near-realtime	many beaches	no

### Video-based optical flow estimation methods

A number of studies have explored the use of video cameras to detect and measure rip currents. Gallop et al. conducted a pilot study to estimate the responses of rip channels to wave energy and wave event duration using a conceptual model based on 40 months of rip channel data obtained from automated analysis of video imagery [14]. Other studies have focused on observing rip current velocity using video recordings of floating tubes placed in the sea at Haeundae Beach, Korea [26,27]. To improve the accuracy of rip current measurements, Stresser et al. recorded videos using a self-stabilizing camera gimbal mounted on a small quadcopter [15]. They used the resulting image sequences to determine spatio-temporal characteristic parameters of the short-term surface wave, and fitted a linear dispersion relationship to the data to monitor the frequency shift caused by the currents. However, while these studies provide valuable insights into the characteristics of rip current features extracted from videos, they were limited to coarse-level detection of rip currents.

The estimation of dense fluid velocity from video has been a topic of interest since the 20th century. Optical flow estimation methods have emerged as an effective approach to address this problem [28,29]. In [30], the integration of wavelets and optical flow estimation methods was proposed for the instant estimation of surface currents from shore-based and UAV videos. Derian et al. developed the “Typhoon” algorithm to extract the apparent displacements (motion) from image sequences specifically for fluid velocity estimation [31]. The algorithm estimates two-dimensional fluid velocity fields from fluid-flow sensory data, such as particle image velocimetry (PIV), schlieren photography, or lidar imagery. The “Typhoon” algorithm was further extended and utilized to estimate the instant surface currents from shore-based and UAV videos in 2017 [12]. The velocity field estimated by the “Typhoon” algorithm was validated by comparison with measurements from an acoustic Doppler velocimeter, demonstrating its ability to capture both wave-to-wave fluctuations and low-frequency variations. The method was also applied successfully to monitor a “flash rip” event. This research demonstrated the potential of optical flow estimation methods for near-shore video analysis.

Classical dense optical-flow methods have well-known failure modes in surf-zone imagery. Illumination changes and specular reflections off wave faces produce spurious motion at the wave-crest level; foam aliasing introduces high-frequency texture that violates the brightness-constancy assumption; turbulent eddies in breaking-wave regions produce flow fields whose pixel-level direction is dominated by wave motion rather than mean current. The temporal-accumulation step in our pipeline is what allows the slow rip-current signal to emerge from this faster, larger-amplitude wave noise.

A separate strand of recent work has applied learning-based optical flow (e.g., FlowNet variants and RAFT [32]) and learning-based rip-current detection to coastal video. Maryan et al. [33] used handcrafted features and a binary classifier to detect rip-current presence at the image level; de Silva et al. [34] trained a CNN on tower-mounted timex (time-exposure) imagery for rip-channel detection. Subsequent work moved to real-time object detectors and larger benchmarks: lightweight YOLO variants for rip localization [35], a rip-current segmentation benchmark with YOLOv8 baselines released at the CVPR NTIRE workshop [36] — the lineage of the RipDetSeg corpus we fine-tune on — a video-instance-segmentation extension of that benchmark [37], and learned anomaly detection on optical-flow pathlines from stationary shore cameras [38]. Of these, the benchmark lineage of [36,37] provides open-source code and released corpora, which is why our deep-learning comparison reproduces exactly that family (a YOLOv8m-seg model fine-tuned on its RipDetSeg successor) on our UAV scenes; the remaining systems [35,38] have no public implementation, so we cite but do not re-implement them. The NTIRE 2026 RipDetSeg challenge [39] released the largest curated rip-current dataset to date (29,307 labeled training images of rip currents) and is the basis for the deep-learning baseline we evaluate against in the Experiment Results section. Our framework differs from these learning-based approaches in three ways: it operates at the pixel rather than image-clip level, it requires no labeled training data for the flow-analysis stage — unlike learned pathline classifiers [38], which need curated pathline training sequences from stationary-camera footage, and it is interpretable in the sense that each pixel’s score is a count of frames in which the optical flow agreed with the locally-estimated offshore direction.

## Proposed rip current detection framework

Fig 1 illustrates the rip current detection framework proposed in this paper based on UAV video analysis. The framework first applies the optical flow estimation method to analyze the UAV video at the frame level and generate a 2-dimensional dense fluid velocity field from a series of adjacent frames. A convolutional neural network and heuristic algorithms are then used to segment the sea water and waves in the frame. Based on these results, the coastline and skyline in the frame can be detected to calculate the offshore direction and fine-grained current velocity for each pixel. These values are aggregated using a novel temporal data fusion method for accurate rip current detection. The proposed framework can produce a likelihood of the existence of rip currents for each pixel in the video.

**Fig 1:**
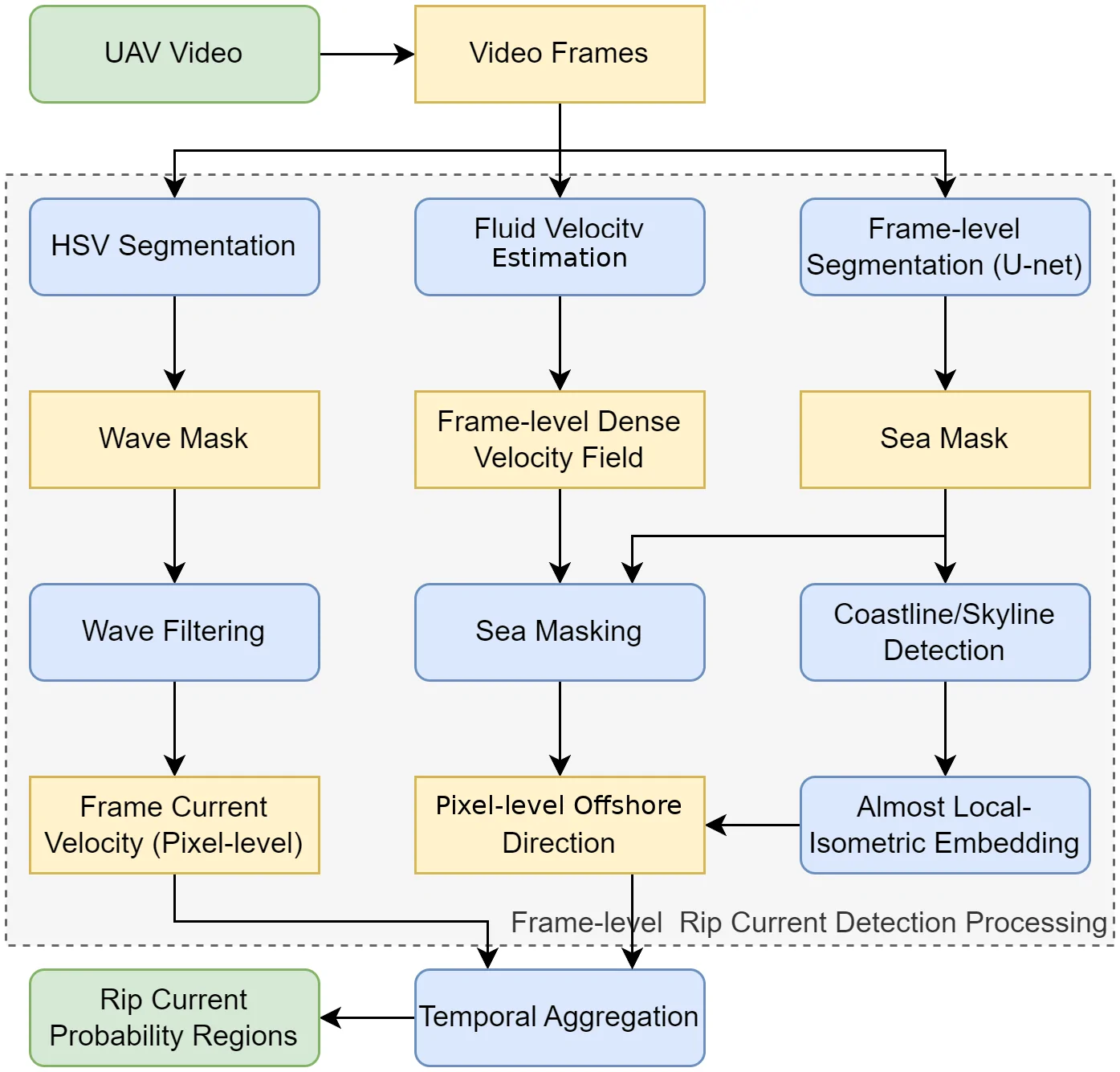
The proposed framework for UAV-video-based rip current detection.

### Fluid velocity estimation methods

In the proposed framework, one of the crucial components for detecting rip currents from UAV videos is to estimate the velocity of seawater. Optical flow estimation methods [28,29], a widely applied technique for video-based velocity estimation, are adopted to produce the two-dimensional velocity field at the fine-grained level. Various optical flow estimation methods are available, and five variants are adopted in this study, which will be discussed in the Experiment Results section. Empirical studies are conducted to evaluate the suitability of these methods for rip current detection. The study revealed that inappropriate constraints of the optimization can adversely affect the detection performance.

#### Optical flow estimation problem formulation.

A two-dimensional velocity field *V* = (*u*, *v*) can be used to describe the optical flow, which represents the pixel-level motions of adjacent frames in a video. The optical flow is determined by minimizing the displaced frame difference (DFD) between a given frame and its leading frame, where *D* is a metric for measuring the difference between the two frames.


$$\begin{array}{c} D=I_{x}u+I_{y}v+I_{t}, \end{array}$$ (1)

where $I_{x}$, $I_{y}$, and $I_{t}$ are the matrices of derivatives of the image intensity values along the *x*, *y*, and time dimensions respectively. In the context of fluid velocity estimation, the estimated velocity field can be regarded as an approximation of the fluid velocity. Equation (2) defines a general formula for such an estimation.


$$\begin{array}{c} \text{arg}\min_{V\in E}F_{d}(V)+\gamma F_{r}(V), \end{array}$$ (2)

where *E* denotes the domain of the two-dimensional velocity field, $F_{d}(V)$ represents the motion modeling term that minimizes DFD, $F_{r}(V)$ is the regularization term for the optimization problem, and γ>0 is the Lagrange multiplier used to balance the motion modeling and regularization terms. Various methods have been proposed to solve this optimization problem and obtain the optical flow estimation.

#### Lucas-kanade optical flow estimation.

One classic method for solving the optical flow estimation problem is the Lucas-Kanade method [29]. This method assumes that the shift of image content is small and approximately constant in a local region between two adjacent frames. Thus, the motion modeling term $F_{d}(V_{t})$ can be defined as:


$$\begin{array}{c} F_{d}(V_{t})=\sum_{i=1}^{W}\sum_{j=1}^{H}D_{i,j}^{2}, \end{array}$$ (3)

where *W* and *H* are the width and height of the frame, $D_{i,j}$ is the DFD value at pixel (*i*,*j*), and no regularization term is applied for Lucas-Kanade method. This assumption is generally correct since the optical flow in successive frames tends to have similar direction and energy.

The Lucas-Kanade method solves this problem by the least-squares principle, yielding the following solution:


$$\begin{array}{c} V=(A^{T}A{)}^{-1}A^{T}b, \end{array}$$ (4)

where $A=[I_{x}^{*},I_{y}^{*}]$, $b=-I_{t}^{*}$, and $I_{x}^{*}$, $I_{y}^{*}$, and $I_{t}^{*}$ are the vectorized derivatives of the image intensity values along the *x*, *y*, and time dimensions, respectively.

#### Horn-schunck optical flow estimation.

Incorporating global constraints for optical flow estimation can improve the performance of the method in scenarios where flow over the entire frame is smooth, as proposed by Horn and Schunck [28]. Thus, the optimization problem in Equation (2) can be modified as follows:


$$\begin{array}{c} \text{arg}\min_{V\in E}\iint D^{2}+\gamma \left({\left|▿u\right|}^{2}+{\left|▿v\right|}^{2}\right)dxdy \end{array}$$ (5)

Unlike the Lucas-Kanade method, which focuses more on local constraints, the Horn-Schunck method takes a holistic and global approach to optical flow estimation. This method is particularly suitable for estimating fluid velocity, as physical constraints must be satisfied in the real world. For instance, detecting flash rip currents, which are not continuous on the ocean surface, can be challenging. The image contents in the local region might not be constant, and the Lucas-Kanade method’s assumption may not always hold in such cases.

#### Typhoon.

In recent years, there has been a growing interest in using wavelet-based methods for optical flow estimation to estimate fluid velocity [31]. One such method is Typhoon, which solves the dense variational optical flow problem by estimating the entire vector field for each pixel of the input images simultaneously [12]. However, compared to traditional motion fields, the DFD term of Typhoon is non-linear; this enables large-displacement estimation but complicates the minimization.

To overcome this challenge, Typhoon incorporates wavelet bases to facilitate the non-linear minimization [12]. By providing a multiscale representation of the motion field, Typhoon can iteratively estimate the movement between images from rough to high precision. In summary, Typhoon is a suitable method for capturing surface waves and currents, especially in the ocean. However, it may not accurately capture rip current optical flow that flashes among waves due to the wavelet-based implementation.

#### High-order regularization (HOR) for optical flow estimation.

Building upon the Typhoon algorithm, we can leverage wavelet bases to improve sensitivity to surface waves and currents. However, this high sensitivity combined with the denoise method can lead to filtering of the majority of the optical flow of rip currents. To address this issue, we incorporate wavelet-based high-order regularization [30] to smooth the estimated flow field and suppress deviations caused by brightness changes and by interfering moving objects such as seagulls.

By combining wavelet-based HOR with primary optical flow estimation methods such as Lucas-Kanade and Horn-Schunck, our algorithm can effectively compute smooth optical flow estimation for most currents on the ocean surface. This estimation enables better rip current detection through our evaluation method.

All experiments use the following solver settings, fixed across all scenes: Lucas-Kanade with a 7×7 integration window; Horn-Schunck with regularization constant α=0.001 and 8 iterations; and the high-order regularized (HOR) variants with a Daubechies-6 wavelet, 3 decomposition levels, and the $\ell_{2}$-norm high-order regularizer, whose gradient fields are passed to the Lucas-Kanade or Horn-Schunck solver respectively. Typhoon runs with the default configuration of its released implementation. All values are recorded in the configuration of the released code.

### Sea water and wave segmentation

As shown in Fig 2A, the UAV videos capture not only the sea surface but also the shore areas, making it necessary to segment out the shore areas to remove possible interference from these environments. However, due to air turbulence and wind, the UAV can be unstable and can shake while recording the videos, resulting in noise on the shore, sky, and some other regions. As a result, the first-order Lucas-Kanade algorithm can produce misleading results.

**Fig 2:**
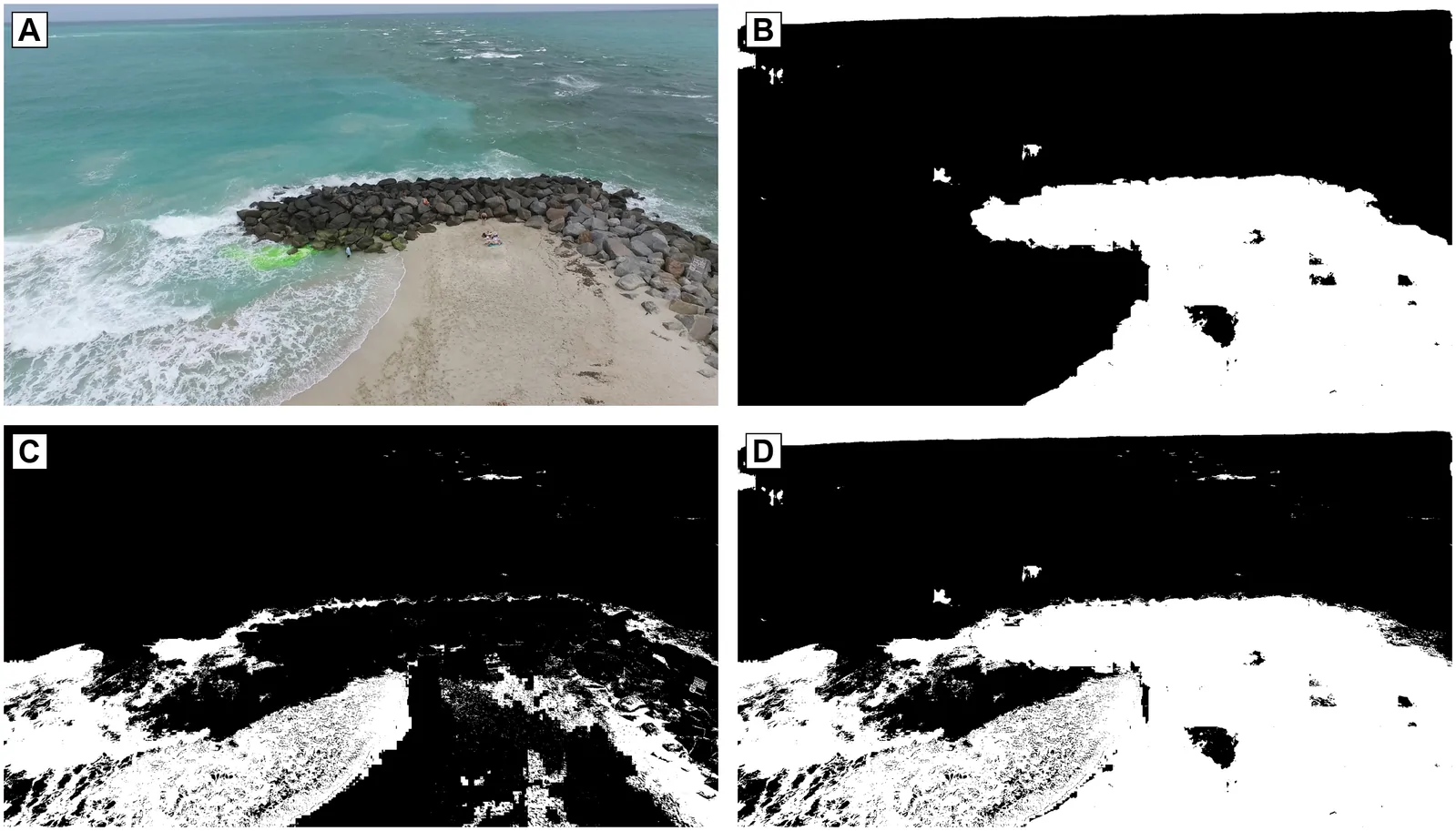
Example of frame-level sea water and wave segmentation. (A) Original frame. (B) Shore-area mask. (C) Wave mask. (D) Combined mask.

Moreover, in UAV videos, the motion of waves on the sea surface dominates the motions captured by optical flow estimation, both because foam-textured wave faces are highly visible to optical flow and because the wave-induced vortex in breaking-wave tails has a direction similar to offshore. The wave-motion signal is therefore noise relative to the slower mean current that defines a rip. We make this distinction explicit: foam is a useful *segmentation feature* (foam pixels mark the sea-side boundary of the surf zone) but the *motion* of foam-textured waves is noise that must be suppressed before the offshore-direction agreement test. The two roles use the same observation (foam visibility) for different downstream purposes.

To segment shore versus sea, we apply a Convolutional Neural Network with a U-Net architecture [40] and a ResNet-18 encoder [41]. The model performs six-class scene parsing (void, sky, sea, land, foam, structure) rather than a binary sea/non-sea decision; this allows downstream stages to treat foam pixels separately when accumulating offshore-agreement counts. The CNN is trained on a custom corpus of 215 distinct UAV frames at 1920×1080 resolution. We note a defect in the released dataset: the accompanying validation split consists of 22 frames that are byte-identical duplicates of frames in the training set, so a genuine held-out split was not preserved when the dataset was packaged. Segmenter quality is therefore quantified below by test-time transfer to scenes absent from training, not by that split. The corpus was assembled from the two *training-only* UAV captures (Ocean Reef Park and South Beach; Table 2), which are separate videos from the two rip-current *evaluation* captures (Palm Beach and Haulover). A second defect of the released dataset belongs here: the per-frame extraction manifest from that assembly was not preserved, so per-capture provenance counts cannot be reconstructed from records. We therefore establish the property the analysis actually depends on — that no training frame is an extraction from either evaluation video — directly rather than by provenance, through the dense frame-matching check reported in the Experiment Results section (subsection “CNN-Refined Sea-Mask Post-Processing”). The combined train+val class pixel distribution is ~38% sky, ~42% sea, ~14% land, ~2% foam, ~2.5% structures (jetties, rocks, debris, people), and ~0.7% void. To achieve fine-grained results we generate up to 50 patches of size 192×216 per training frame at stride 96×108 (10,150 training patches in total); validation and inference use 200 patches per frame at stride 48×54 with majority voting. Sea-segmentation IoU and F1 scores — on the held-out validation frames and, as test-time transfer, on the two never-seen evaluation scenes — appear in Table 3; the shore-area mask is shown in Fig 2B.

**Table 2:** Summary of near-shore UAV video metadata.

Video name	Altitude	Location	Start time	Features of the area	Rip current
Palm Beach	50 m	26.793436, −80.031638	3:34 PM, April 27, 2016	Clear water, rock outcropping	Captured
Haulover	70 m	25.900867, −80.121667	2:09 PM, April 6, 2016	Complex rocky jetty	Captured
Ocean Reef Park	Varying	26.792321, −80.032065	9:07 AM, June 18, 2016	Clear water, rock outcropping	Not captured
South Beach 1	Varying	26.792321, −80.032065	1:11 PM, Sept. 29, 2018	Sargassum seaweed present	Not captured
South Beach 2	45 m	25.791486, −80.127193	11:51 AM, Feb. 21, 2021	Bar gap present	Captured
YouTube videos	NA	NA	NA	NA	Captured

**Table 3:** Sea-segmentation quality of the six-class segmenter. The segmenter is trained on the scene-parsing corpus assembled from the training-only captures (Ocean Reef Park and South Beach); dense pixel-matching confirms that no frame from either evaluation video is in its training or validation set (verified in the “CNN-Refined Sea-Mask Post-Processing” subsection). The first row is an *in-sample* measurement: the released validation split duplicates training frames exactly, so it does not estimate generalization and is reported only for completeness. The last two rows are the segmenter’s *test-time transfer* to the two evaluation scenes it never saw, evaluated by full-frame argmax against each scene’s static sea mask; these are the generalization estimates.

Evaluation setting	IoU (sea)	F1 (sea)
In-sample (duplicated validation split)	0.728	0.843
Palm Beach — test-time transfer (unseen)	0.484	0.653
Haulover — test-time transfer (unseen)	0.575	0.730

To remove residual wave-motion noise within the sea region, we additionally generate a wave mask using foam-color thresholds in HSV space (the “wave mask” panel of Fig 2C). We acknowledge that fixed HSV ranges are a heuristic and not robust across all lighting conditions or sediment-laden water; future work could replace this step with the YOLOv8m-seg segmentor evaluated in the Experiment Results section, which was trained on a much larger and more visually diverse RipDetSeg dataset. The combined mask in Fig 2D is the union of the shore-area and wave masks; the offshore-direction estimation in the next section uses this combined mask to restrict the analysis to “calm” sea pixels.

### Off-shore direction calculation

Assuming a resolution of n×m for a frame containing the distance between each pixel point (*x*,*y*) and the shoreline, the directional derivative can be calculated using the gradient of the distance matrix to determine the direction of motion for each pixel. By treating the calculated horizontal gradients (*u*) as rows and vertical gradients (*v*) as columns, a vector can be created for each pixel on the ocean surface. Using camera projection in 3-D space, the direction of each point on the ocean surface can be accurately determined in real-world coordinates by mapping from a spherical planet to 2-D frames. This is achieved through near-local isometric embedding techniques on the sphere, with the shoreline and skyline providing reference points to assess the range of directions offshore. Through this pipeline, it is possible to determine whether a pixel on the ocean surface has an off-shore direction. The proposed pipeline can be simplified and divided into the following steps during implementation.

#### Almost local-isometric embedding modeling on sphere.

We map each image-plane point onto the Earth’s spherical ocean surface and construct a projection that is *almost local-isometric* at a chosen observation point. As an overview of the construction: with the camera at altitude *h* above a sphere of radius *R* and pose (θ,ψ), each screen point *p* = (*a*,*u*,*v*) defines a ray whose intersection with the sphere is the smaller root of a quadratic in the ray parameter *t*; the sign of the discriminant separa*t*es screen points that see the ocean surface from those that see the sky (the skyline). Projecting the visible region *D* onto the sphere yields a map *f*, and composing it with a spherical-coordinate chart $g_{q_{0}}$ centered at an observation point *q*_0_ gives $h=g_{q_{0}}\circ f:D\to {\mathbb{R}}^{2}$. Because no map from a spherical cap to the plane can preserve every pairwise distance, we choose *q*_0_ to minimize a total distance-distortion functional *W*(*q*) (or its squared variant $W^{\prime }(q)$), which makes *h almost* local-isometric — distances from *q*_0_ are preserved exactly and general pairwise distances are minimally distorted. The per-pixel Jacobian of *h* maps image-plane velocity to ground-plane velocity and, at strict nadir, reduces to the isotropic scaling (h/f)·I — with *f* the focal length, played by the screen distance *a* in the derivation below — that preserves angles exactly; its detection contribution and the quantitative planar-versus-sphere bound are given in the Sphere-Projection Contribution subsection of the Results. The full construction follows.

Assume the screen is located at (*a*,0,0) with size n×m; the coordinates of the four corners are $\left(a,\pm \frac{n}{2},\pm \frac{m}{2}\right)$.

The distance between the camera and the ground is *h* and the radius of the earth is *R*.

Assume the center of earth *E* is


$$\begin{array}{c} \left((h+R)\cos(\theta )\sin(\psi ),(h+R)\sin(\theta )\sin(\psi ),(h+R)\cos(\psi )\right) \end{array}$$

The equation of earth *E* is


$$\begin{array}{c} E:(x-(h+R)\cos(\theta )\sin(\psi ){)}^{2}\\ +(y-(h+R)\sin(\theta )\sin(\psi ){)}^{2}\\ +(z-(h+R)\cos(\psi ){)}^{2}=R^{2} \end{array}$$ (6)

and assume cos(θ)sin(ψ)>0, i.e., the *x*-coordinate of the center of the earth *E* is positive.

Pick a point *p* on the screen *p* = (*a*,*u*,*v*) with $-\frac{n}{2}\leq u\leq \frac{n}{2},-\frac{m}{2}\leq v\leq \frac{m}{2}$, and there is a line passing through the camera and *p*:


$$\begin{array}{c} l(t)=(at,ut,vt) \end{array}$$ (7)

The intersection points of the line with the earth is given by the following equation:


$$\begin{array}{cc} & (at-(h+R)\cos(\theta )\sin(\psi ){)}^{2}+\\ & (ut-(h+R)\sin(\theta )\sin(\psi ){)}^{2}+\\ & (vt-(h+R)\cos(\psi ){)}^{2}=R^{2} \end{array}$$ (8)

After simplification, we have a quadratic equation in *t*,


$$\begin{array}{cc} & (a^{2}+u^{2}+v^{2})t^{2}-2(h+R)(a\cos(\theta )\sin(\psi )+\\ & u\sin(\theta )\sin(\psi )+v\cos(\psi ))t+h(h+2R)=0 \end{array}$$ (9)

To solve for *t*, we first compute the discriminant of this quadratic equation.


$$\begin{array}{cc} \Delta = & 4(h+R{)}^{2}(a\cos(\theta )\sin(\psi )+u\sin(\theta )\sin(\psi )+\\ & v\cos(\psi ){)}^{2}-4(a^{2}+u^{2}+v^{2})h(h+2R) \end{array}$$ (10)

If we let Δ=0, it gives the equation of the skyline


$$\begin{array}{c} C=\{\Delta =0\} \end{array}$$

and we should propose conditions $-\frac{n}{2}\leq u\leq \frac{n}{2},-\frac{m}{2}\leq v\leq \frac{m}{2}$ to restrict it to the screen.

Assume the line *l* intersects with the earth, i.e., Δ≥0, then


$$\begin{array}{cc} t= & \frac{2(h+R)\left(a\cos(\theta )\sin(\psi )+u\sin(\theta )\sin(\psi )+v\cos(\psi )\right)}{2(a^{2}+u^{2}+v^{2})}\\ & \pm \frac{\sqrt{\Delta }}{2(a^{2}+u^{2}+v^{2})} \end{array}$$ (11)

The point we could see corresponds to


$$\begin{array}{cc} t(u,v)= & \frac{2(h+R)}{2(a^{2}+u^{2}+v^{2})}(a\cos(\theta )\sin(\psi )+u\sin(\theta )\sin(\psi )\\ & +v\cos(\psi ))-\frac{\sqrt{\Delta }}{2(a^{2}+u^{2}+v^{2})} \end{array}$$ (12)

We have a map *f* from domain


$$\begin{array}{c} D=\{(u,v):u\in [-\frac{n}{2},\frac{n}{2}],v\in [-\frac{m}{2},\frac{m}{2}],\Delta \geq 0\} \end{array}$$ (13)

to the earth *E* given by


$$\begin{array}{c} f:(u,v)\to (at(u,v),ut(u,v),vt(u,v))=p(u,v) \end{array}$$ (14)

where *t*(*u*,*v*) is given as above.

To calculate velocity, we need the distance between two points on earth *E*, obtained from their images on the screen.

We are looking for a local isometry I:U→V where *U* is an open neighborhood of a point *p* in the screen and V⊂E is an open neighborhood of *f*(*p*) such that


$$\begin{array}{c} d(q_{1},q_{2})=d_{c}(f(q_{1}),f(q_{2}))\;\forall q_{1},q_{2}\in U \end{array}$$

where *d* is the Euclidean distance on the screen and $d_{c}$ is the great-circle distance on the earth.

However, the separation of the image points on the screen does not correspond to the great-circle distance between the original points on Earth. The reason is that no local isometry exists between the curved surface of the earth and the flat screen. A local isometry must preserve curvature, but the screen has curvature 0, while a sphere of radius *R* has curvature 1/*R*^2^.

Let us study the great-circle distance $d_{c}$ on a sphere of radius *R*.

Given two points $q_{1}=(x_{1},y_{1},z_{1})$, $q_{2}=(x_{2},y_{2},z_{2})$ on sphere $S:x^{2}+y^{2}+z^{2}=R^{2}$, the great-circle distance is


$$\begin{array}{c} d_{c}(q_{1},q_{2})=R\cdot \arccos\;\left(\frac{q_{1}\cdot q_{2}}{R^{2}}\right) \end{array}$$ (15)

We could write the Euclidean coordinates into spherical coordinates with the chosen north pole *q*_0_ = (0,0,*R*), as the observation point, and 0 longitude angle and for a general point q∈S, we have *q* = (*x*,*y*,*z*) where


$$\begin{array}{cc} & x=R\cos(\phi )\sin(\delta )\\ & y=R\sin(\phi )\sin(\delta )\\ & z=R\cos(\delta ) \end{array}$$ (16)

and write down the map $g_{q_{0}}$ from the $S_{z\geq 0}$ to plane ${\mathbb{R}}^{2}$ where *q*_0_ is the north pole.


$$\begin{array}{cc} g_{q_{0}}(x,y,z) & =g_{q_{0}}(R\cos(\phi )\sin(\delta ),R\sin(\phi )\sin(\delta ),R\cos(\delta ))\\ & =(R\cdot \delta \cdot \cos(\phi ),R\cdot \delta \cdot \sin(\phi )) \end{array}$$ (17)

The map


$$\begin{array}{c} |g_{q_{0}}|(x,y,z)=|(R\cdot \delta \cdot \cos(\phi ),R\cdot \delta \cdot \sin(\phi ))|=R\cdot \delta \end{array}$$

does not depend on the choice of 0 longitude angle. Moreover, if we pick a different 0 longitude angle, it is equivalent to rotation $S_{z\geq 0}$ by some angle and rotation does not change the Euclidean distance or the great-circle distance. Consequently, once a point *q* is fixed, the function *W*(*q*) (to be defined later) is invariant under rotations about *q*. This is why we need not specify the 0 longitude angle when we define function $g_{q_{0}}$.

We notice


$$\begin{array}{c} d_{c}(q,q_{0})=R\cdot \delta =d(g_{q_{0}}(q),g_{q_{0}}(q_{0})),\;\forall q\in S_{z\geq 0}, \end{array}$$

which means the distance between an arbitrary point *q* and the observation point *q*_0_ is preserved under map $g_{q_{0}}$. But for a general pair of points $q_{1},q_{2}\in S_{z\geq 0}$, the distance between them is not preserved,


$$\begin{array}{c} d_{c}(q_{1},q_{2})\neq d(g_{q_{0}}(q_{1}),g_{q_{0}}(q_{2})) \end{array}$$

We may translate the same metric to *E* and define function


$$\begin{array}{c} W(q)=\iint_{f(D{)}^{2}}|d_{c}(q_{1},q_{2})-d(g_{q}(q_{1}),g_{q}(q_{2}))|dq_{1}dq_{2} \end{array}$$ (18)

where q∈f(D), $d_{c}$ is the great-circle distance and *d* is the Euclidean distance.

Since *f*(*D*) is closed and bounded and *W* is continuous, *W* attains its absolute minimum at some point $q_{0}\in f(D)$. We find the optimal observation point *q*_0_ such that the total difference between the distances *d* and $d_{c}$ is minimal.

We may also consider other functions like


$$\begin{array}{c} W^{\prime }(q)=\iint_{f(D{)}^{2}}|d_{c}(q_{1},q_{2})-d(g_{q}(q_{1}),g_{q}(q_{2})){|}^{2}dq_{1}dq_{2} \end{array}$$ (19)

to find the optimal observation point *q*_0_.

With initial data a,h,R,θ,ψ, we compute the optimal observation point *q*_0_ and construct a map $h=g_{q_{0}}\circ f:D\to {\mathbb{R}}^{2}$ that is “almost local-isometric”, since we pick the optimal observation point to minimize the function *W*(*q*).

#### The estimation of local offshore direction.

After obtaining an almost local-isometric projection of videos onto the actual Earth’s ocean surface, we introduce a pipeline designed to identify shoreline information within the projected almost local-isometric frame masks. Gaussian filtering is used to reduce noise in the mask, followed by the application of the Canny edge detector to obtain the edge image from the mask. Subsequently, the coastline is determined using the detected edge image. If the coastline is discontinuous or outside the edge image boundary, the bottom edge of the image is assumed to be part of the coastline for subsequent calculations. Once the shoreline information is detected, we can formulate a matrix *S* that represents the distances between each pixel in the frame and the detected shoreline points $\left(N_{1},M_{1}),(N_{2},M_{2}),...,(N_{n},M_{n}\right)$ as follows:


Si,j=min{(N1−i)2+(M1−j)2,(N2−i)2+(M2−j)2,=...,(Nn−i)2+(Mn−j)2} (20)

Using the distance matrix *S*, we can compute the potential flow in the frame. The results are then masked with the generated shore area mask to obtain the vectors of pixel points on the sea surface in the potential flow, which are referred to as offshore directions $OV_{i,j}$. The offshore direction matrix based on the segmentation masks can be visualized as a flow map, as shown in Fig 3.

**Fig 3:**
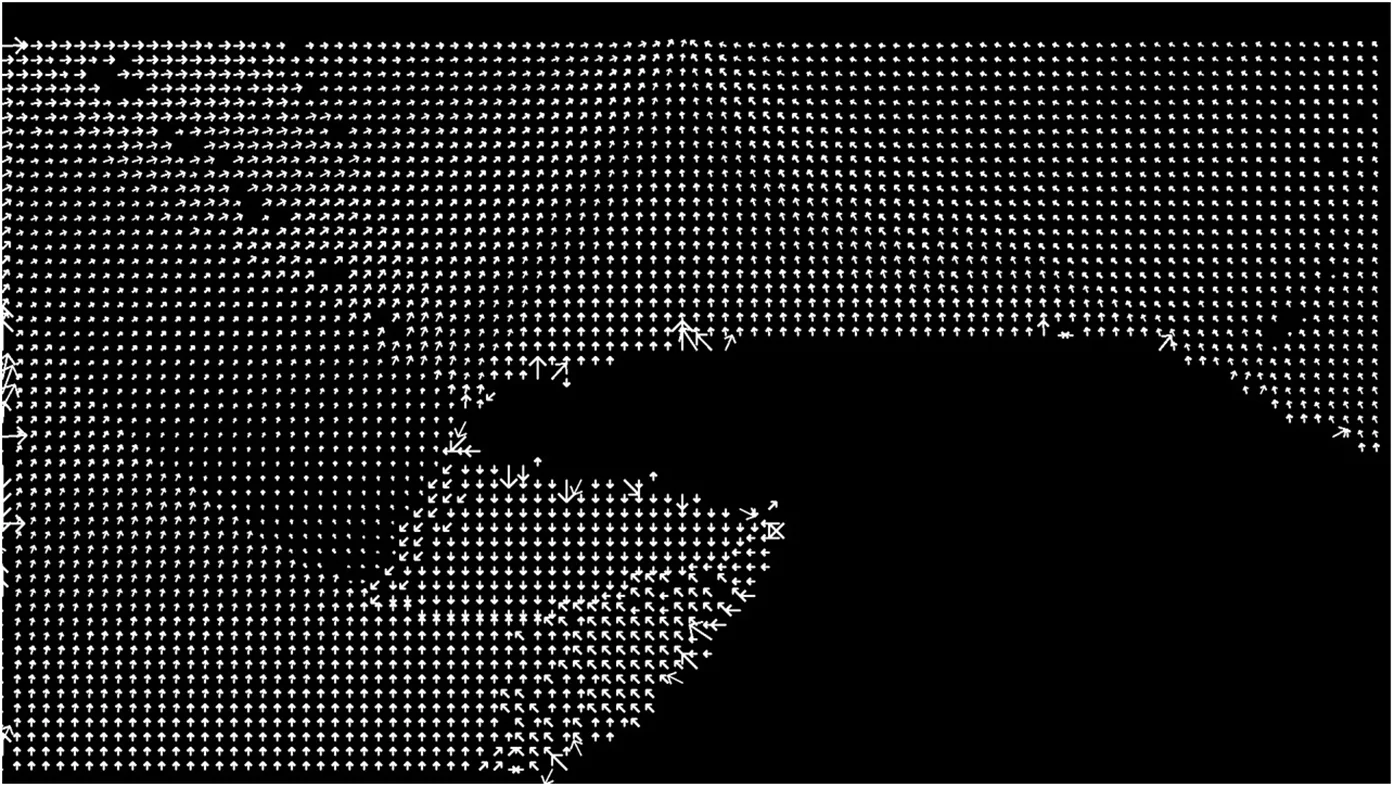
Pixel-level offshore-direction field, visualized as a flow map.

### Rip current detection

Even when the estimated current velocity and offshore direction are obtained, it is hard to evaluate whether there is a rip current using a single frame. Therefore, temporal data fusion is introduced to utilize the estimated velocity fields over time. For the *t*-th frame $I_{t}$ in the video, let $V_{t,i,j}=(u_{t,i,j},v_{t,i,j})$ be the estimated velocity field using any optical flow estimation method, $M_{t}^{g}$ be the ground mask, and $M_{t}^{c}$ be the combined mask. Given the offshore direction $OV_{t,i,j}$, the rip currents can be defined as any current directed away from the seashore, with the angle between the current direction and $OV_{t,i,j}$ within the range (−T,+T), where *T* is the angular tolerance. The default $T=90^{\circ }$ is not a fitted hyper-parameter but the physical boundary of seaward-directed flow: a velocity vector has a positive component along the offshore direction — i.e., it is directed away from the shore rather than toward it — if and only if it lies within $\pm 90^{\circ }$ of $OV_{t,i,j}$. The offshore direction, not the coastline, is the reference; $T=90^{\circ }$ therefore admits exactly the seaward half-plane, and $T<90^{\circ }$ is the tunable *restriction* that additionally rejects obliquely-seaward flow. Because real rip necks are frequently curved or angled relative to the shore normal [42,43], a narrower tolerance risks discarding genuine rip flow; we therefore adopt the neutral $T=90^{\circ }$ as the default. This choice is also robust empirically: taking the median of raw per-clip AUC across the 16 South Beach sub-clips, $T=90^{\circ }$ is the median-best threshold for both HOR Horn-Schunck and HOR Lucas-Kanade (on the per-scene-normalized median reported in the Angular Tolerance Sensitivity subsection of the Experiment Results, by contrast, smaller tolerances score higher; the two statistics answer different questions and we report both; the mean-based statistic, included in the released results, selects $15^{\circ }$ for HOR Horn-Schunck). Across these sub-clips the median AUC spans only ~0.006 over the whole nine-point sweep, close to the chance level set by their 1.6–9.6% rip-pixel prevalence, so the sub-clip evidence discriminates weakly among tolerances and the choice of *T* rests on the physical argument above rather than on it, and PR-AUC is insensitive to *T* across a broad band on 18 UAV scenes (the Experiment Results section reports the full sensitivity sweep over $T\in \{15^{\circ },30^{\circ },45^{\circ },60^{\circ },75^{\circ },90^{\circ },105^{\circ },120^{\circ },150^{\circ }\}$).

We have an almost local-isometric map $h=g_{q_{0}}\circ f:D\to {\mathbb{R}}^{2}$ from the initial setting (context distinguishes this map from the flight altitude *h* of the sphere model), and this map *h* is differentiable and injective. Given a vector field *V* on domain *D*, it will induce a vector field *dh*(*V*) on *h*(*D*) where


$$\begin{array}{c} \left(dh(V))(h(p))=dh_{p}(V(p)\right) \end{array}$$ (21)

Given two vector fields $OV_{t,i,j}$ which is offshore direction, and $V_{t,i,j}$ which is pixel optical flow measurement on *D*, the angle between $dh(OV_{t,i,j})$ and $dh(V_{t,i,j})$ at point *h*(*p*) is given by


$$\begin{array}{c} \Theta (h(p){)}_{t,i,j}=arccos(\frac{dh_{p}(OV_{t,i,j}(p))\cdot dh_{p}(V_{t,i,j}(p))}{\left|dh_{p}(OV_{t,i,j}(p))|\cdot |dh_{p}(V_{t,i,j}(p))\right|}) \end{array}$$ (22)

where p∈D. Then we can compare $\Theta (h(p){)}_{t,i,j}$ with the tolerance *T* (default $90^{\circ }$) to detect whether this pixel in this frame is moving offshore.

Since various factors could cause fluid velocity towards the offshore direction, it is unreliable to estimate the rip current regions based on the results of a single frame. Thus, to fuse the frame-level data into a stable, fine-grained estimation of rip current regions, we aggregate the temporal information over a window of *N* frames (we reserve *T* for the angular tolerance). This is accomplished by generating a rip-current frequency heatmap through a process of temporal accumulation.

First, a count matrix *L*, with the same dimensions as the video frames, is initialized with zeros.

Then, the matrix is recursively updated for each frame. For every pixel (*i*,*j*) at each time step *t*, we check if it is part of an offshore-directed flow characteristic of a rip current. If it is, the pixel’s corresponding value in the count matrix is incremented. This process is defined by the following recursive formula:


$$\begin{array}{c} L_{t,i,j}=\left\{ \begin{array}{cc} L_{t-1,i,j}+1 & \text{if pixel }(i,j)\text{ is moving offshore at frame }t\\ L_{t-1,i,j} & \text{otherwise} \end{array}\right. \end{array}$$ (23)

where $L_{t,i,j}$ represents the accumulated count for pixel (*i*,*j*) over the time period [1, *t*], with the initial condi*t*ion *L*_0,*i*,*j*_ = 0 for all pixels. The condition “pixel (*i*,*j*) is moving offshore at frame *t*” is met if the flow-direction devia*t*ion satisfies $\Theta (h(p){)}_{t,i,j}<T$, with the default $T=90^{\circ }$. The accumulated $L_{N,i,j}$ is therefore a per-pixel count of frames in which the optical-flow direction agreed with the offshore reference; we refer to this quantity as the rip-current frequency score (rather than “likelihood”) to avoid implying a calibrated probability; the phrase “likelihood map” is retained elsewhere only as the informal name of the output product.

After iterating through all *N* frames, the final matrix $L_{N}$ contains the frequency count for each pixel. To generate the final normalized heatmap *P*, we divide each element by the window length *N*:


$$\begin{array}{c} P_{i,j}=\frac{L_{N,i,j}}{N} \end{array}$$ (24)

The resulting value $P_{i,j}$ lies in [0,1]; a higher value indicates more temporally-persistent offshore-directed flow at that location, which is the signature of a rip current.

**Why temporal accumulation suppresses wave noise.** Optical flow measures the *apparent* motion of visible surface texture (foam, dye, and sediment fronts acting as tracers), not the water velocity directly. It is therefore essential to state what the accumulation step does to the error between the two. We model the observed per-pixel flow as


$$\begin{array}{c} V_{t,i,j}=v_{i,j}^{\text{rip}}+v_{t,i,j}^{\text{wave}}+\varepsilon_{t,i,j}, \end{array}$$ (25)

the sum of (i) a slow, temporally-persistent seaward drift *v*^rip^ that defines a rip; (ii) an oscillatory wave-orbital / breaking-wave component *v*^wave^ that is quasi-periodic and, over an integer number of wave periods, has zero temporal mean; (iii) tracer noise ε from foam formation, glint, and estimation error, approximately zero-mean but short-range temporally correlated (foam patches and glint persist on the order of a second); and (iv) — important because it bounds what this method can ever reject — a possible temporally-persistent *non-rip* seaward component *v*^other^ such as undertow, wind-driven surface drift, or bathymetrically channelled wave-driven flow. The accumulation step is an indicator average: pixel (*i*,*j*) accrues a count in each frame in which its flow direction lies within ±T of the offshore direction. For a persistent rip, the direction test is satisfied in nearly every frame, so the count grows ∝N with the number of accumulated frames *N*. For the zero-mean terms the test outcome decorrelates on the wave-period timescale τ, not frame-to-frame: at a 30 fps effective rate and a ~6 s period, the 600-frame window contains only $N_{\text{eff}}\approx N/(\text{fps}\cdot \tau )\sim 3$–10 effectively independent samples, so the noise contribution behaves as a random walk of scale $\propto \sqrt{N_{\text{eff}}}$ and the rip-to-noise ratio grows $\propto \sqrt{N_{\text{eff}}}$ — coherent integration of the persistent component, limited by temporal correlation. This is the behaviour reported in the Temporal Accumulation Suppresses Wave Noise subsection of the Experiment Results: rapid gain over the first few wave periods, then saturation. Two limitations of the accumulator follow directly from the model: it cannot reject the persistent seaward term *v*^other^, which is scored identically to a rip (the high-scoring non-rip region over the shallow outcropping in the Palm Beach results is an observed instance); and it cannot integrate indefinitely, because under residual UAV motion a fixed image pixel samples drifting ground locations, which eventually blurs the accumulator (the short-window generalization subsection of the Results).

## Experiment results

### Near-shore UAV video data acquisition

We present a dataset of four UAV-recorded videos of the near-shore area in the United States, three of which capture rip currents (Palm Beach, Haulover, and South Beach; only the first two serve as primary evaluation scenes) (the South Beach capture comprises two segments, listed separately in Table 2). All videos were captured at 60 fps using a DJI Phantom 3 Advanced Quadcopter UAV, equipped with a Full HD 1920×1080 camera; the pipeline processes every second frame (30 fps effective) and a 3-axis stabilization gimbal to reduce image blurring. Prior to processing by the optical flow estimation methods, all video frames were converted to gray-scale. Table 2 provides metadata for the videos.

One of the two videos that captured a rip current was recorded at Riviera Beach, located in Palm Beach County, Florida. This beach features a structurally-controlled rip current that forms through an outcropping of coquina limestone rock called the Anastasia formation. At low tides, the rock outcropping is almost fully exposed, and fluorescein dye delineates the rip current as it flows seaward through the rocks. The flow characteristics of this rip current showed a relatively straight-flowing rip that terminates at the outer edge of the surf zone. The video was captured on April 27th, 2016, during good weather conditions, with significant wave height of 0.6 m from the east-southeast and wind speeds of only 10 kts from the east [44,45].

The other rip current video was recorded at Haulover Park, which is the largest surf beach park in Miami-Dade County and is situated north of Miami Beach. The park features a jetty structure with a beach-parallel spur (breakwater) at the end that acts as a barrier against waves. Rip currents form in response to longshore currents generated by obliquely-approaching waves from the northeast. The southward-flowing longshore current is deflected by the jetty to form a structurally-controlled rip that flows to the north and then offshore. The video was recorded on April 6th, 2016, in good weather conditions, with significant wave height of 0.8 m, spilling breakers, and a 6 second period, and wind speeds of 15 kts from the northeast [44,45].

The other two videos in our dataset were recorded at Ocean Reef Park and South Beach, both in Florida, and were used for training the proposed sea-segmentation CNN model and contribute no frame to the Palm Beach and Haulover headline evaluation; the South Beach captures also serve as generalization test sub-clips (angular-tolerance sweep and short-window evaluation); because those captures also supplied segmenter training frames, the seg-gated sub-clip results are not scene-independent, as disclosed in the corresponding section. To increase the diversity of video frames in the dataset, the UAV’s location, pose, and flight altitude varied throughout the recordings. All four videos showcase different types of near-shore scenes, where the color of the sea water, beach, and rock, the objects in the near-shore areas, and the shape of the shores differ. Our proposed framework is limited to videos with clear vision of the sea surface and near-shore area, recorded under good weather conditions. However, in the future, we plan to extend our work to include videos captured under different weather conditions, such as rainy and stormy days.

Data Acquisition and Compliance: The four primary UAV videos were provided by researchers from the Department of Earth & Environment at Florida International University, with permission granted for their use in this study; the flights were conducted by that research team. Supplementary video data was sourced from public posts on YouTube. The collection and analysis methods for all data complied with the terms and conditions of the original sources. All footage was recorded over public beach areas from altitudes of 45–70 m, at which individual beachgoers are not personally identifiable; no identifiable individuals appear in any figure, and no personal data were collected or analyzed, so this study did not require institutional review board approval.

### Fluid velocity estimation performance evaluation

#### Qualitative assessment via quiver plots.

The quiver plot is a useful technique for visualizing two-dimensional velocity fields and aiding oceanographers in observing estimated fluid velocities. To generate a quiver plot based on the estimated velocity *V* of a frame, the frame is initially split into blocks of size $N_{W}\times N_{H}$, where the size of the frame W×H is divided into $\frac{W}{N_{W}}\times \frac{H}{N_{H}}$ blocks. For each block, an average velocity is computed using the equation:


$$\begin{array}{c} {\bar{V}}_{block}=\frac{1}{N_{W}N_{H}}\sum_{i=1}^{N_{W}}\sum_{j=1}^{N_{H}}V_{i_{0}+i,j_{0}+j}, \end{array}$$ (26)

where $V_{i,j}$ is the velocity at pixel (*i*,*j*), $\left(i_{0},j_{0}\right)$ represents the index of the pixel in the left-upper corner of the block, and it is assumed that $\frac{W}{N_{W}}$ and $\frac{H}{N_{H}}$ are integers.

Once the average velocity for each block is computed, an arrow is superimposed on the frame image for each block, where the length of the arrow represents the speed, and the direction of the arrow indicates the direction of the velocity. An example of a quiver plot is shown in Fig 4.

**Fig 4:**
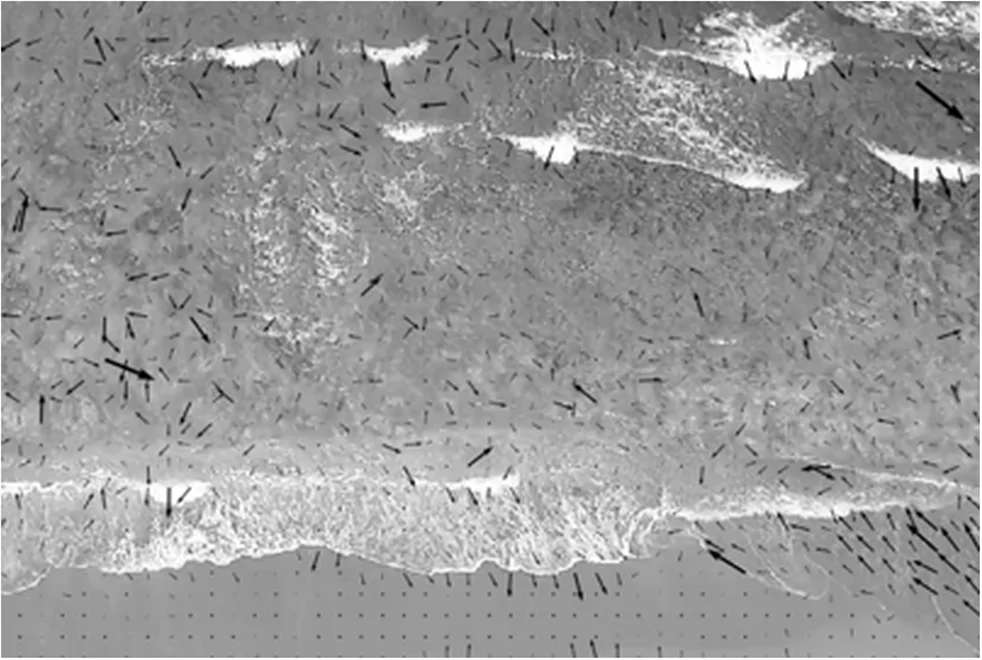
Example quiver plot for the Palm Beach video.

To visualize the velocity estimation and evaluate whether the estimated fluid velocity aligns with the actual rip current direction as measured in the field, quiver plots for all adjacent frames in the video are generated and concatenated to produce a quiver plot video. In the case of the Haulover and Palm Beach videos in the experiment, the Typhoon optical flow estimation method’s quiver plot results are favored because it provides a cleaner velocity estimation compared to other algorithms. However, it should be noted that the Typhoon method tends to ignore the velocity of subtle currents and provides only an estimate for the waves with foam, which are visually identifiable.

#### Virtual drifter.

In rip current research, dye is commonly used to detect rip currents. To qualitatively evaluate the velocity estimation results of our proposed framework, we conducted a virtual drifter experiment. The experiment is designed based on the virtual drifter experiment used in [12]. In the experiment, fluorescein (yellow) dye was released into the water, as shown in Fig 5.

**Fig 5:**
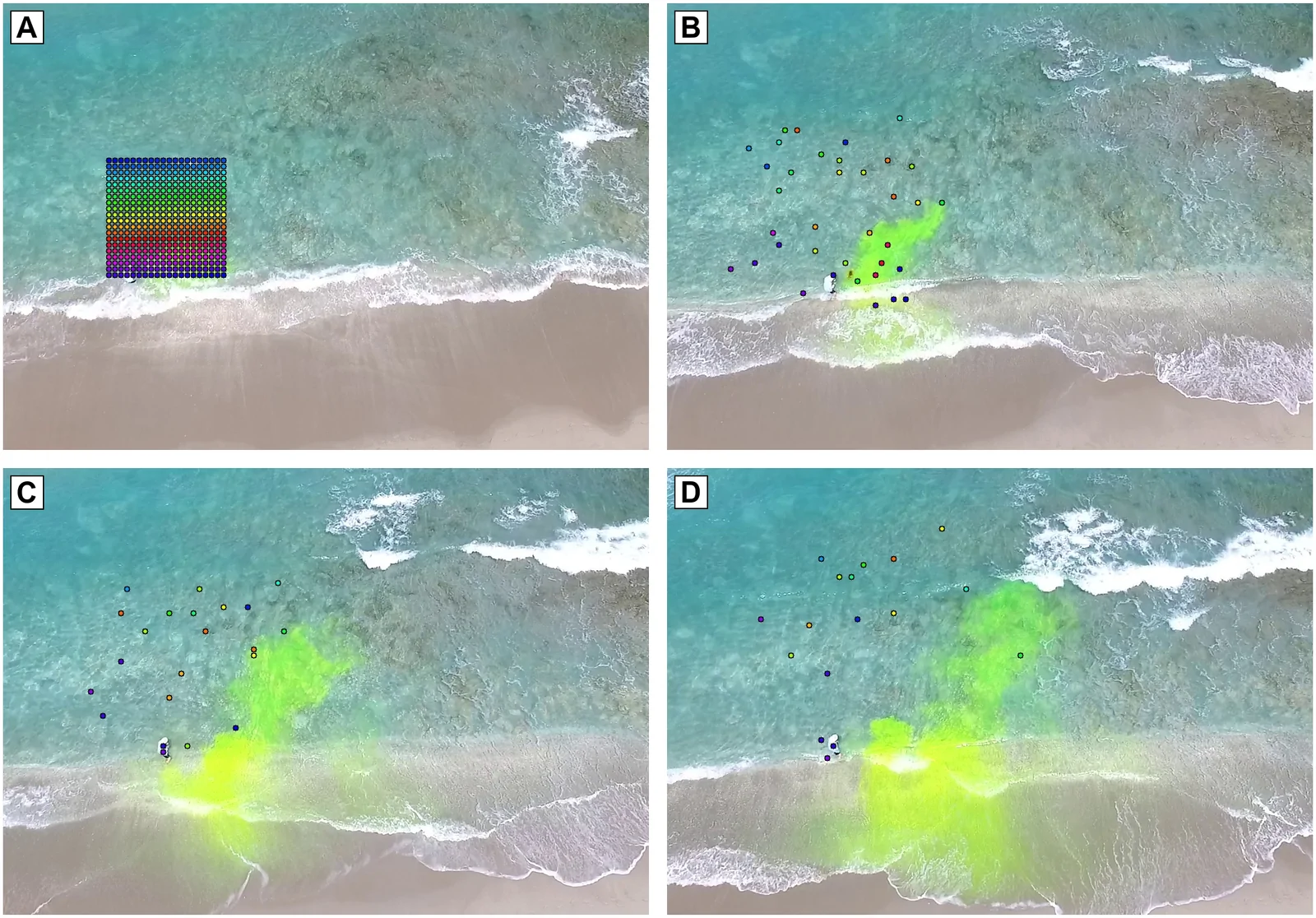
Positions of virtual drifters for the Palm Beach video. (A) Initial positions. (B) After 20-second simulation. (C) After 40-second simulation. (D) After 60-second simulation.

We densely generated 400 virtual drifters in a 200×200 rectangular box with equal spacing and superimposed them on the original frame. The region of dye was thoroughly covered by the virtual drifters. As the velocity at each pixel can be obtained based on our proposed framework, we simulated the changes in virtual drifters’ locations by the estimated velocity at the drifter locations. The positions of the virtual drifters were tracked for 20, 40, and 60 seconds, as shown in Fig 5B–D. The results demonstrated that the virtual drifters followed the path of the rip current accurately despite outpacing the speed of the dye. The drifter trajectories are thus consistent with the observed dye transport, supporting the estimated velocity field (as noted in the Conclusion, this is a consistency check internal to the same velocity field, not an independent validation). However, it was also observed that the virtual drifters can be significantly affected by surface waves, indicating the importance of removing wave interference for rip current detection.

### Sea water segmentation performance evaluation

The six-class scene-parsing segmenter is trained on 215 distinct frames assembled from the two training-only captures (Ocean Reef Park and South Beach; Table 2); as noted in the Methods, the per-frame extraction manifest was not preserved, so their disjointness from the evaluation videos is established by dense matching rather than by provenance records. The released validation split of 22 frames duplicates training frames exactly (Methods), so the IoU on that split is an in-sample measurement; we report it below only as such, and rely on test-time transfer for generalization; as established in the “CNN-Refined Sea-Mask Post-Processing” subsection, no frame of either rip-current evaluation video is present in this set. Training and inference use the patch protocol described in the Methods.

Table 3 reports the segmenter’s sea-class IoU and F1. On the released validation split it attains IoU 0.728, but because that split duplicates training frames this is an in-sample figure rather than a generalization estimate. Because the two rip-current *evaluation* scenes are never seen during training, we report the segmenter’s quality on them as *test-time transfer*: applying the trained segmenter to sampled frames of each evaluation video and comparing the predicted sea region to that scene’s static sea mask, it attains IoU 0.484 (Palm Beach) and 0.575 (Haulover). As expected for out-of-training scenes, transfer IoU is lower than the in-distribution validation IoU; it is nonetheless sufficient for the soft *P*(sea) gate, whose downstream detection contribution is quantified — and shown to be leakage-free — in the CNN-refinement subsection.

### Rip current detection performance evaluation

The proposed rip current detection framework scores each pixel by its accumulated offshore-agreement frequency. To establish a clear performance benchmark, we compare five optical-flow variants within the same pipeline: Lucas-Kanade, Horn-Schunck, their high-order regularized (HOR) variants, and Typhoon. Fig 6 illustrates the per-algorithm heatmaps on both the Haulover and Palm Beach videos, with higher accumulated scores shown in hotter colors; all results in this experiment use a 20-second accumulation window. The heatmaps show characteristic qualitative differences: the Horn-Schunck family, which leverages global regularization, produces spatially smoother heatmaps whose high-score regions align well with the annotated rip channels; the Lucas-Kanade family produces sharper but noisier responses with lower contrast against the background; and Typhoon’s wavelet regularization suppresses many subtle currents. Quantitatively, however, no single algorithm dominates across scenes — plain Lucas-Kanade attains the strongest per-algorithm AUC on Palm Beach while the Horn-Schunck family leads on Haulover (Table 4). This scene-dependent, algorithm-level diversity is precisely what the cross-algorithm ensemble of the Spatial Smoothing and Cross-Algorithm Ensemble subsection exploits: at its per-scene-selected operating point the ensemble dominates every individual algorithm on both scenes (at the neutral $T=90^{\circ }$ default it is comparable to the strongest single algorithm on Palm Beach).

**Table 4:** Precision-recall AUC for both UAV scenes. Each scene’s per-algorithm column reports each optical-flow algorithm’s natural per-scene baseline: for Palm Beach, the seg-gated patched pipeline at the per-algorithm optimal angular tolerance $T_{⋆}=150^{\circ }$ (full sweep in Table 5); for Haulover, the recovered output of the original 2020 pipeline (uint8-reinterpreted from the int8-truncated stored arrays, masked to the static sea mask). These two baselines reflect each scene’s native per-algorithm configuration: seg-gating helps Palm Beach (where the rip channel has visually distinct sea texture) but is roughly neutral on Haulover (where rip-vs-non-rip *P*(sea) contrast is near zero). The bottom row is the full pipeline (CNN gating + Gaussian smoothing σ=25 px + normalized cross-algorithm ensembling). Its headline operating point is the physically-neutral default $T=90^{\circ }$, at which it attains 0.636 (Palm Beach) and 0.660 (Haulover). The bottom-row values shown with *T* selected per scene to maximize ensemble AUC (0.7066 / 0.7040) are an upper bound obtained by selecting on the evaluation annotation itself, and are labelled as such throughout (Tables 6 and 7).

Optical Flow Estimation Method	Palm Beach	Haulover
Typhoon [12]	0.660	0.234
Lucas-Kanade	0.686	0.216
HOR Lucas-Kanade	0.616	0.467
Horn-Schunck (HS)	0.640	0.396
HOR Horn-Schunck	0.640	0.480
**Full pipeline, neutral default $T=90^{\circ }$**	**0.636**	**0.660**
Full pipeline, per-scene-selected (upper bound)	0.7066	0.7040

**Fig 6:**
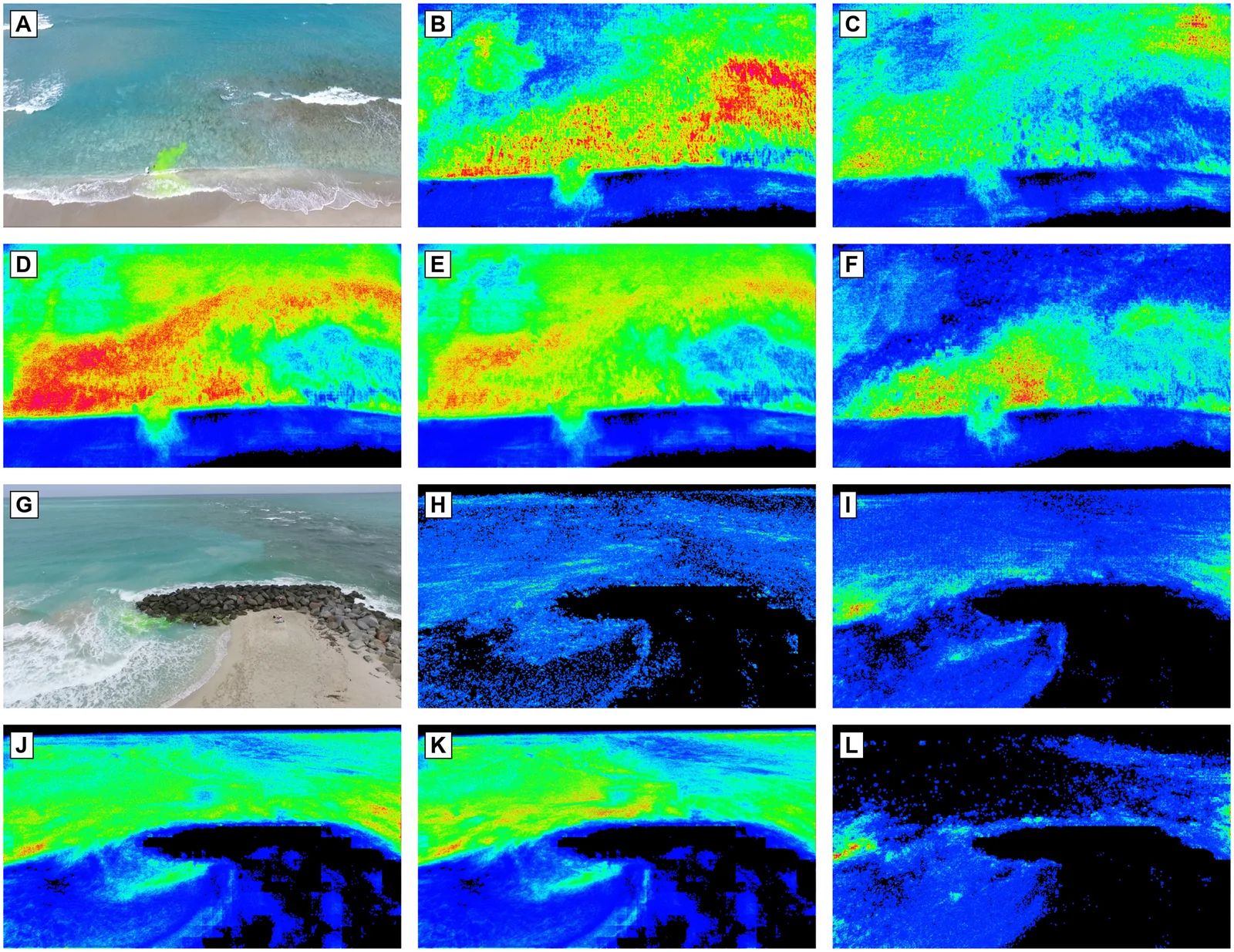
Rip-current frequency heatmaps for the Palm Beach (A–F) and Haulover (G–L) videos using the five optical-flow estimation methods. (A, G) Original video frames. (B, H) Lucas-Kanade. (C, I) HOR Lucas-Kanade. (D, J) Horn-Schunck. (E, K) HOR Horn-Schunck. (F, L) Typhoon [12].

The Palm Beach video has a large oceanic area, with the sea taking up a significant portion of the frame; however, over the rock outcropping the water is shallow enough that the seabed is visible through it, which introduces potential seabed-texture noise into the optical flow in the right-middle part of the frame. Overall, the heatmaps contain two high-scoring regions. The lower-left region corresponds to the expert-confirmed rip current; a second high-scoring region appears in the upper right, where wave-driven flow over the shallow outcropping partially mimics offshore-directed motion.

In this study, we evaluate the effectiveness of our proposed rip-current detection framework by comparing its output against expert ground truth (Fig 7). Ground-truth annotations are produced through consensus among three coastal-science domain experts and provided to us by Dr. Stephen B. Leatherman; the consensus process is intended to resolve, not bypass, inter-annotator disagreement on rip-current boundaries. We quantify the underlying disagreement explicitly in the short-window generalization analysis, where two independent expert annotations are available per sub-clip (mean Cohen κ=0.254). For per-pixel evaluation we compute precision-recall curves (Fig 8); we use precision-recall AUC as the headline metric rather than pixel accuracy because the positive class is the minority everywhere: annotated rip pixels cover 28.3% of frame pixels at Palm Beach and 11.2% at Haulover, and only 1.6–9.6% on the South Beach sub-clips. These fractions are also the expected PR-AUC of a random scorer, so the neutral-default headline results of 0.636 and 0.660 exceed chance by factors of 2.2 and 5.9 respectively (the per-scene-selected upper bounds 0.7066/0.7040 by 2.5 and 6.3).

**Fig 7:**
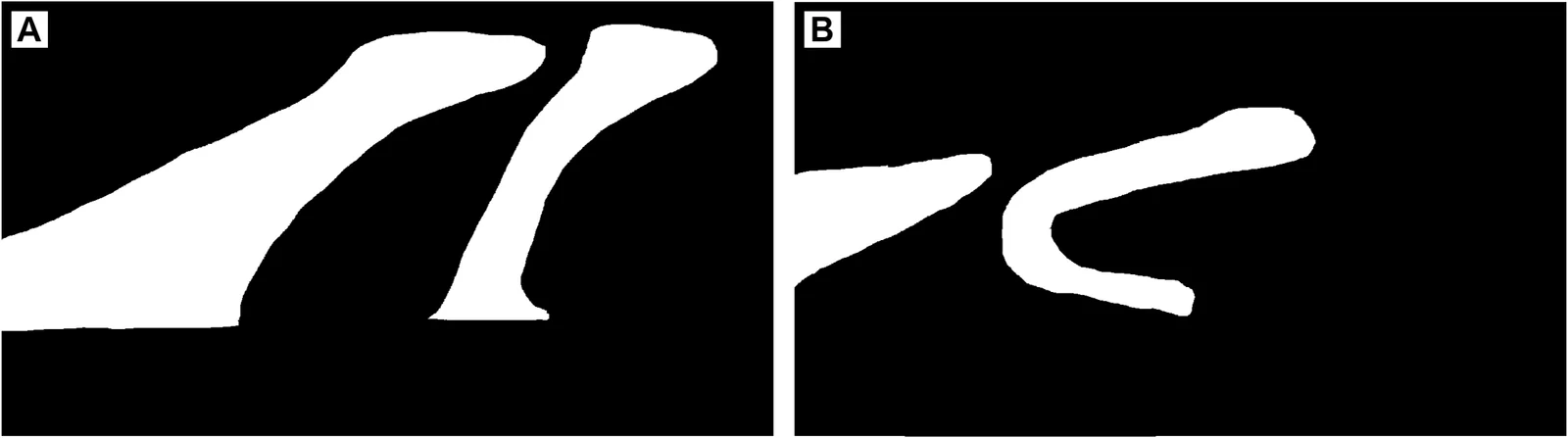
Expert rip current region annotations for both videos. (A) Palm Beach video. (B) Haulover video.

**Fig 8:**
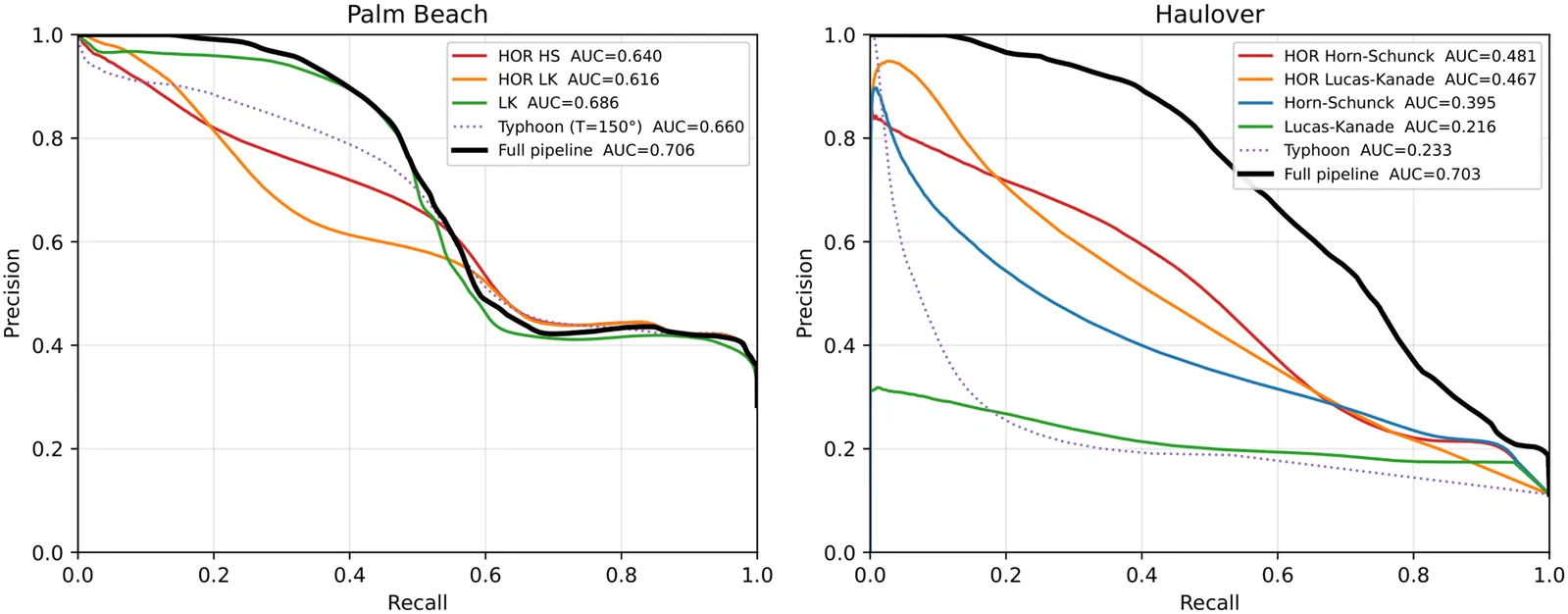
Precision-recall curves for the three seg-gated optical-flow algorithms (HOR Horn-Schunck, HOR Lucas-Kanade, Lucas-Kanade), the canonical Typhoon [12] baseline, and the full smoothed-ensemble pipeline (bold black) on Palm Beach (left) and Haulover (right). Each curve label includes the per-curve PR-AUC. Per-algorithm curves use each scene’s configuration from Table 4: the seg-gated pipeline at $T=150^{\circ }$ for Palm Beach, and the canonical 2020 output (natively computed at the $T=90^{\circ }$ criterion) for Haulover. The full-pipeline curve uses each scene’s ensemble operating point ($T=150^{\circ }$ for Palm Beach; the canonical ensemble plus the $T=15^{\circ }$ seg-gated LK term for Haulover); at that per-scene-selected operating point — shown here as an upper bound, not the neutral-default headline — it dominates every individual algorithm on both scenes.

We define the region of rip current at a given level of acceptance *a* as $R^{a}=\{(i,j)|L_{N,i,j}\geq a\}$, where $L_{N,i,j}$ is the accumulated frequency score estimated by our framework for the pixel at (*i*,*j*) and *a* is the threshold. The precision and recall values at the pixel-level are then computed as follows:


$$\begin{array}{c} {\text{precision}}^{a}=\frac{\left|R^{a}\cap R_{gt}\right|}{\left|R^{a}\right|},{\text{recall}}^{a}=\frac{\left|R^{a}\cap R_{gt}\right|}{\left|R_{gt}\right|} \end{array}$$ (27)

where |·| refers to the number of pixels in the given region and $R_{gt}$ is the region of rip current based on the expert’s annotation. The curve is traced over a dense uniform grid of score thresholds and integrated with the trapezoidal rule; the exact procedure ships in the released evaluation code. We report PR-AUC as a point estimate: for a fixed scene, window, and annotation the metric is a deterministic functional of every evaluated pixel, so there is no sampling variability to bootstrap, and pixel-level resampling would be invalid because neighbouring pixels are strongly spatially correlated. The relevant uncertainty is instead across scenes, operating points, and annotators, which we report directly: the 18-scene angular-tolerance sweep, the per-sub-clip AUC distribution (0.15–0.63 across the 16 South Beach sub-clips; 0.10 and 0.11 on the two supplementary YouTube clips, so 0.10–0.63 over all 18), the accumulation-length and smoothing sensitivity analyses, and the inter-annotation Cohen κ of 0.254. With two primary evaluation scenes, formal hypothesis testing across scenes would be underpowered and is not attempted.

We observe that the precision-recall curves for the different optical-flow estimation methods exhibit distinct patterns. Precision typically increases as recall decreases, indicating that more accurate results can be obtained when accepting fewer rip-current regions; where precision instead falls at low recall, high-scoring noise has been admitted as rip current. For instance, the Lucas-Kanade curves are affected by noise in areas with rocky seabeds (the right part of the Palm Beach frame), and all per-algorithm curves sit substantially lower on Haulover than on Palm Beach, reflecting that scene’s weaker rip-to-background contrast. No single algorithm’s curve dominates on both scenes — consistent with the per-algorithm AUC ordering of Table 4 — whereas the full smoothed-ensemble pipeline (bold black in Fig 8; per-scene-selected operating point) dominates every individual algorithm on both.

Table 4 reports the area under the precision-recall curve (PR-AUC) for each individual optical-flow algorithm at its native per-scene baseline together with the full-pipeline result on both videos. On Palm Beach the per-algorithm AUCs cluster between 0.61 and 0.69, with plain Lucas-Kanade strongest at $T=150^{\circ }$. On Haulover the HS-family algorithms lead (HOR Horn-Schunck at 0.480, Horn-Schunck at 0.396) because the original 2020 pipeline’s per-frame motion mask filters out most non-rip flow, and the HS regularizer recovers smoother spatial agreement under that mask. The cross-algorithm ensemble in the bottom row is invariant to the per-scene per-algorithm ordering: each algorithm’s smoothed heatmap is max-normalized before averaging, and the average dominates every individual algorithm by a large margin on both scenes at its per-scene-selected operating point; at the neutral default the margin narrows (Table 4).

It should be noted that in the right part of the Palm Beach video, the detected rip current region is separated into two streams, whereas the expert annotation covers the entire area as one. Since there is no dye released in this area, it is impossible to validate the real pattern of the rip current regions. Nevertheless, the rip current has been clearly identified and matched with the expert’s annotation. In addition, the rip currents in the middle-bottom part of the Haulover video have almost reverse direction compared to the others, which violates the basic assumption of Eq. 22. As a result, the framework might fail to detect rip currents when there is no constant offshore direction in the video. In the case of a complex coastline, the framework is limited in its ability to identify the correct offshore direction and may thus produce incorrect rip current detection results.

### Angular tolerance sensitivity

As established at its definition, $T=90^{\circ }$ is the physical boundary of seaward-directed flow (the offshore half-plane), not a value fitted to maximize AUC. Here we document empirically that detection performance is moreover *insensitive* to *T* over a broad band, so the result does not depend on a finely-tuned tolerance. We first sweep $T\in \{15^{\circ },30^{\circ },45^{\circ },60^{\circ },75^{\circ },90^{\circ },105^{\circ },120^{\circ },150^{\circ }\}$ on the Palm Beach scene, computing PR-AUC at each *T* against the same annotation used for Table 4 (Fig 9); both HOR variants stay within 0.05 AUC of their peak across the entire $T\in [75^{\circ },150^{\circ }]$ band.

**Fig 9:**
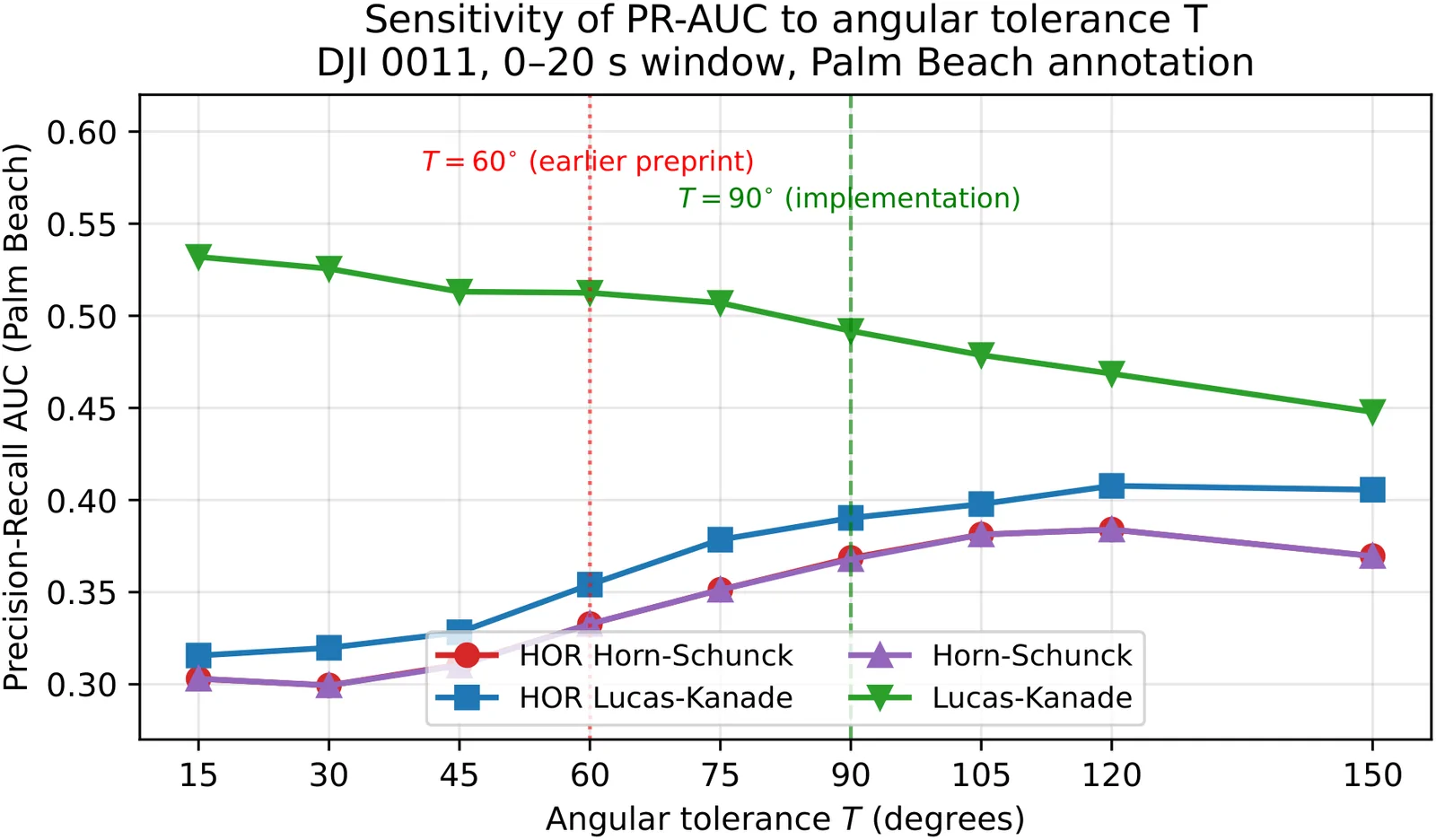
PR-AUC vs the angular tolerance *T* on the Palm Beach scene (0–20 s window matching the expert annotation). Both HOR variants peak at $T\in [105^{\circ },120^{\circ }]$ and are within 0.05 AUC of their peak across the entire $T\in [75^{\circ },150^{\circ }]$ band. The default $T=90^{\circ }$ choice (vertical dashed line) is within 0.05 AUC of the peak for both algorithms. We adopt $T=90^{\circ }$ — the physical boundary of seaward-directed flow — as the framework default.

To show this is not a single-scene artifact, we extend the sweep to 18 UAV scenes (Palm Beach, Haulover, and 16 South Beach sub-clips), all with the same optical-flow algorithm (HOR Horn-Schunck, no CNN gating) for a fair comparison (Fig 10). The median normalized PR-AUC is 0.878 at $T=90^{\circ }$ and stays within [0.838, 0.881] across the whole $T\in [60^{\circ },150^{\circ }]$ band — a broad plateau rather than a sharp peak. The per-scene optima are spread from $15^{\circ }$ to $150^{\circ }$: there is no single tolerance that maximizes AUC across scenes. We therefore adopt the physically-neutral $90^{\circ }$ as the framework default and report it as the headline operating point. Where a per-scene optimum is also reported, it is labelled as such: those values are selected against the same expert annotation used for evaluation and are consequently an optimistic upper bound rather than an expected-performance estimate.

**Fig 10:**
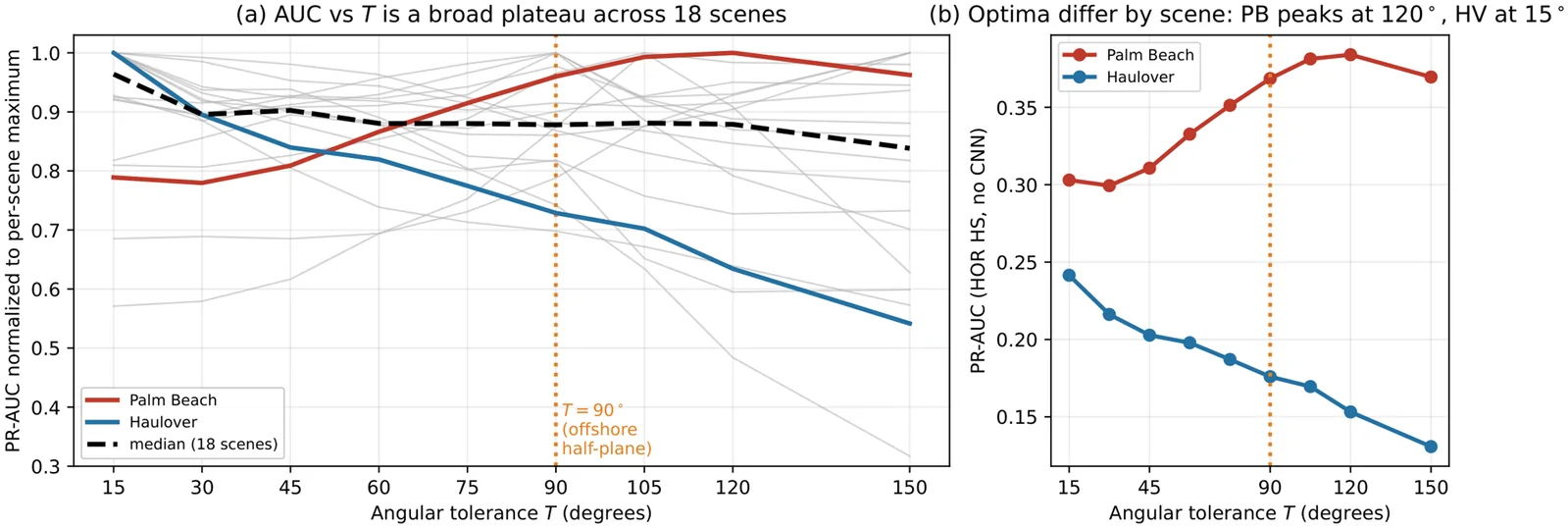
Angular-tolerance sensitivity across 18 UAV scenes (HOR Horn-Schunck, no CNN gating). (a) PR-AUC normalized to each scene’s own maximum versus *T*; individual scenes (grey), the two evaluation scenes (colored), and the 18-scene median (dashed) all form a broad plateau, and $T=90^{\circ }$ (the offshore half-plane; dotted line) sits within it. (b) Raw PR-AUC on the two evaluation scenes: Palm Beach rises with *T* to a peak at $120^{\circ }$ while Haulover falls monotonically and favors the smallest tolerance swept, illustrating that no single tolerance is universally optimal and that the neutral $90^{\circ }$ default is AUC-maximizing on neither.

**How much does the direction test contribute?** Because the Palm Beach ensemble AUC rises monotonically with *T* up to $150^{\circ }$ — the largest tolerance swept — we ran the corresponding degenerate control: the identical seg-gated, σ=25-smoothed pipeline with the direction test *removed entirely*, so that every moving sea pixel contributes regardless of direction. That control attains PR-AUC 0.618 on Palm Beach, against 0.636 at the neutral $T=90^{\circ }$ default and 0.707 at $T=150^{\circ }$. Three conclusions follow, and they qualify our second contribution. First, on this scene the majority of the full-pipeline score is carried by segmentation gating and spatial smoothing, not by direction consistency: the neutral-default direction test adds only + 0.018 over no direction test at all. Second, the direction test is nonetheless not inert — at its best setting it adds + 0.088 — but the useful criterion on Palm Beach is the permissive one, which rejects only flow within $30^{\circ }$ of *directly onshore*, rather than the strict one requiring strongly seaward flow. Third, this mechanism explains the monotone trend rather than leaving it as an unexplained artifact: tightening *T* toward $90^{\circ }$ progressively discards obliquely-seaward rip flow, which on a structurally-controlled rip with a curved neck is a substantial fraction of the true signal. We report the neutral default as the headline because the alternative is a per-scene selection against the evaluation annotation. (The same control cannot be run on Haulover: its canonical accumulators are archived 2020 arrays with the $T=90^{\circ }$ criterion already applied during accumulation, so the direction test cannot be removed retroactively; the qualification above is therefore established for Palm Beach only.)

Throughout the paper we therefore report *both* operating points: at the neutral $T=90^{\circ }$ default the full pipeline attains 0.636 (Palm Beach) and 0.660 (Haulover), versus 0.7066 / 0.7040 at the per-scene optima of the fully-disclosed sweep (Tables 6 and 7), keeping the default-versus-tuned distinction explicit.

### Temporal accumulation suppresses wave noise

To verify the noise-suppression argument of the Methods (“Why temporal accumulation suppresses wave noise”), we measure PR-AUC as a function of the accumulation length, from a single frame up to the full 20-second window, on both evaluation scenes at $T=90^{\circ }$ with no CNN gating (so the effect isolates accumulation alone). Fig 11 shows the predicted behavior: a single-frame direction map is noise-dominated (AUC 0.09–0.26), and accumulating over just 1–3 seconds roughly *doubles* the AUC (to 0.20–0.49) as the zero-mean wave component averages out. The Haulover curve here is a from-scratch re-run, so its absolute level (HOR HS 0.176 at 20 s) sits well below the archived 2020 canonical value of Table 4 (0.480), which was produced by the original system from optical-flow data that was not retained; only the *shape* of the curve, not its level, is interpreted in this subsection. Performance then saturates near 3–5 seconds and declines toward the full window — by up to 16% relative for HOR Horn-Schunck on Palm Beach (0.437 at 3 s versus 0.369 at 20 s) — consistent with correlation-limited averaging compounded by residual UAV motion (drift was measured at 5–10 px/s on the South Beach clips; the primary scenes’ drift is quantified in the Fig 11 caption). The headline results nonetheless use the full 20 s window: the expert annotations were produced against the full clips, and selecting a shorter window post hoc for its higher AUC would be another selection against the evaluation annotation, which we avoid throughout. The single-frame-to-accumulated gain is the direct empirical signature that per-frame optical flow is wave-noise-dominated and that accumulation extracts the persistent rip signal.

**Fig 11:**
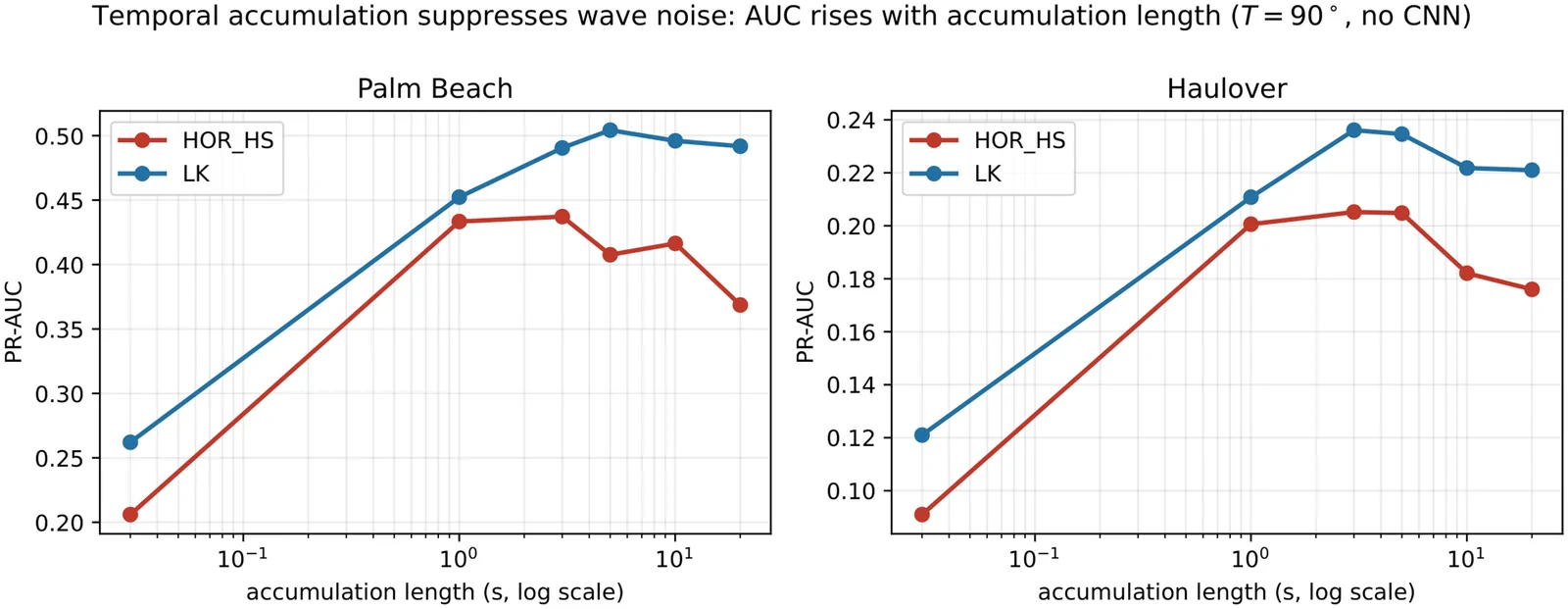
PR-AUC versus temporal-accumulation length (log scale) on Palm Beach and Haulover, at $T=90^{\circ }$ with no CNN gating. On both scenes a single frame is noise-dominated and accumulation over 1–3 s roughly doubles the AUC before saturating; the post-peak decline reflects correlation-limited averaging and UAV motion; phase-correlation cumulative drift over the 20 s window measures ≤23 px on Haulover but reaches ~230 px mid-window on Palm Beach. This is the empirical counterpart of the correlation-limited coherent-integration argument in the Methods.

### CNN-refined sea-mask post-processing

A per-frame learned sea mask provides finer rejection of land, structure, and breaking-wave foreground than a static manually-drawn mask. We trained a U-Net segmenter (ResNet-18 ImageNet-initialized encoder, 14.3 M parameters) for 30 epochs on the scene-parsing training set (215 training + 22 validation frames at 1920×1080, six-class BGR-bit-encoded labels: void, sky, sea, land, foam, structure), using AdamW at learning rate 10^–4^ with weight decay 10^–4^, batch size 4, class-weighted cross-entropy loss, 512×512 random crops with horizontal flips, and a cosine learning-rate schedule; the checkpoint with the best sea-class IoU on the released validation split is retained (an in-sample criterion, since that split duplicates training frames; Table 3). The training script and checkpoint are in the released repository. On that same split the trained model attains a full-frame IoU of 0.728 on the sea class, 0.736 on land, and 0.523 on foam — in-sample figures; the held-out generalization estimates are the test-time transfer IoUs of Table 3. The pipeline then multiplies the per-pixel angular-agreement indicator **1**[angle < *T*] by the U-Net’s per-frame sea probability P(sea∣frame) before the temporal accumulation; we call this multiplicative factor the *sea-gate*, and heatmaps produced with it *seg-gated*. The streaming pipeline holds peak resident memory below 4 GB by computing one frame’s segmenter output at a time and never materializing the full per-frame probability stack.

**No training-frame leakage from the evaluation videos.** We verify directly that the segmenter was never exposed to any frame of the two rip-current evaluation videos. For each of the 215 distinct scene-parsing frames we compute the minimum pixel-wise mean-squared error (MSE) against a dense sample of each evaluation video (96×54 luminance, every second frame). A genuine extraction would yield MSE ≈0; instead the minimum MSE over all 215 training frames is 674 (on the 0–255^2^ scale, i.e., an RMS difference of ≈26 grey levels), with median 1726 and *no* frame below 200. The Haulover video was searched across its full duration (all 24,576 frames, every second frame; at 60 fps consecutive frames are near-identical, so this covers every distinct view). The Palm Beach video is decodable up to frame 15,395 (257 s) — after which its bitstream is irrecoverably corrupt with every decoder tested — so it was searched across that span, which contains the entire 0–20 s evaluation window (and 12× beyond). Within every searched span no training frame is an extraction from either evaluation capture, and no frame approaches an extraction (minimum MSE 674). A simpler nearest-neighbour assignment, which labels each training frame by whichever of the two evaluation videos it is closer to, splits the corpus 139/76 (Haulover / Palm Beach); because every frame is forced onto one of the two videos regardless of true origin, and the “nearest” frame is always ≥674 MSE away, that split is a forced two-way assignment, not provenance (it is used below only to select the frames removed in the leakage-controlled retraining). The released match table makes the degeneracy concrete: of the 139 frames the matcher assigns to Haulover, 134 are assigned to the *same* frame of that video — an out-of-focus ground-level shot recorded while the aircraft was on the sand — at a minimum MSE of ≥674. A statistic in which a large majority of assignments collapse onto one degenerate frame cannot evidence scene overlap. Accordingly the leakage-controlled retraining below removes the frames that matcher flags, which is the conservative reading of the leakage argument, rather than a set selected for scene similarity; the matched random-exclusion control is what isolates training-set size. The corpus was assembled from the separate Ocean Reef Park and South Beach captures (Table 2); as noted in the Methods the per-frame extraction manifest was not preserved, so the property established here is the one the analysis depends on — disjointness from the evaluation videos — rather than positive provenance. The CNN is in any case trained only on scene-parsing labels, never on rip-current labels.

**Frame-level disjointness is not scene-level disjointness.** We distinguish two claims, because only the first is established. *Frame- and video-level disjointness* is proven above: no training frame is an extraction from either evaluation capture. *Scene-level disjointness is not claimed, and the coordinates in* Table 2 *argue against it*: the Ocean Reef Park training capture lies approximately 131 m from the Palm Beach evaluation site and shares its described setting (clear water over a rock outcropping). The segmenter was therefore trained on imagery of substantially the same beach, recorded on a different day, even though it saw no frame of the evaluation video itself. Readers should interpret the CNN gate’s Palm Beach contribution with that in mind. Two further observations follow from this proximity. First, an auxiliary deep-embedding attribution that we release assigns 88 of the 215 distinct training frames to an evaluation scene as nearest neighbour (97 of the 237 released files, which include the 22 duplicates); this is expected rather than contradictory, because Ocean Reef Park was not among its candidate sources, so its frames necessarily fall to the visually near-identical Palm Beach capture. Second, the effect can be measured directly: as reported next, removing the training frames most similar to Palm Beach costs no more accuracy than removing the same number of frames at random. Two limitations of this analysis remain. No frame-extraction manifest was preserved when the training set was built, so per-capture provenance counts cannot be reconstructed — a dense pixel-MSE search against every available source video (both evaluation captures, all 16 South Beach sub-clips, and the two auxiliary captures) finds no extraction match for any training frame (minimum MSE 448), which establishes video-file-level disjointness from everything we can search but leaves the original capture assignment undocumented. For the same reason a capture-level holdout (retraining with the entire Ocean Reef Park capture excluded) cannot be constructed reliably; the similarity-based exclusion plus the matched-random control above are the closest available substitute, and we identify the capture-level holdout as the definitive experiment should the original extraction records be recovered.

**The CNN gate’s contribution is generalization, not memorization.** Because no evaluation-scene frame is present in training, the reported result is already free of test-scene leakage. As a further robustness check we retrained the six-class segmenter twice, each time *removing every training frame that the two-way nearest-neighbour matcher assigns to the held-out evaluation scene* – i.e., the same frames that a leakage argument would flag – and re-ran the seg-gated pipeline on that scene. Even with those frames removed (76 of the 215 distinct images, 35%, for the Palm Beach retraining; 139 images, 65%, for Haulover), CNN gating still improves Palm Beach over the ungated baseline at every algorithm: peak per-algorithm PR-AUC rises from 0.384 to 0.458 (HOR Horn-Schunck) and from 0.532 to 0.602 (Lucas-Kanade), versus 0.640 and 0.686 for the full-data segmenter. The residual gap to the full-data model is attributable to the smaller training set rather than to scene familiarity, and we test that attribution directly with a matched control: retraining with 76 *randomly chosen* training images removed — the same number the similarity-based exclusion removes — costs essentially the same accuracy. Peak Palm Beach PR-AUC falls from 0.640 (full data) to 0.491 under random removal and 0.458 under similarity-based removal for HOR Horn-Schunck, and from 0.686 to 0.579 and 0.602 respectively for Lucas-Kanade; averaged over the two algorithms the drops are −0.128 (random) and −0.133 (similarity-based), and for Lucas-Kanade removing the *most similar* frames is actually less damaging than removing random ones. Training-set size, not exposure to the evaluation scene, therefore accounts for the gap. The gap cannot reflect test-set memorization, since no evaluation-video frame is present in any of these training sets. On Haulover the sea-gate does not help — it is roughly neutral for the Lucas-Kanade term (0.273→0.278 leakage-free) and *reduces* the HS-family AUC by 46% relative (0.242→0.131) — consistent with the near-zero rip-vs-non-rip *P*(sea) contrast on this scene noted above; accordingly the sea-gate is not used in the Haulover headline, which is built from the canonical heatmaps and post-processing described below. As an additional fully-external reference, a binary segmenter trained only on the public RipDetSeg corpus — with zero contact with any scene in this study — used as the gate attains a peak per-algorithm PR-AUC of 0.604 on Palm Beach (Lucas-Kanade; full sweep in the released CSVs), independently confirming that a gate benefiting Palm Beach requires no exposure to the evaluation scenes. The frame-matching script and the leakage-controlled checkpoints are released with the code.

Table 5 reports the resulting AUC on Palm Beach across the angular-tolerance sweep for HOR Horn-Schunck, HOR Lucas-Kanade, plain Horn-Schunck, and plain Lucas-Kanade. The CNN refinement substantially boosts the per-algorithm AUC; the strongest single algorithm (Lucas-Kanade at $T=150^{\circ }$) reaches 0.686. The CNN gate’s contribution comes from suppressing spurious flow activations on sand, structure, and breaking-wave foreground that a static mask cannot exclude per-frame.

**Table 5:** Palm Beach per-algorithm AUC after CNN-refined per-frame sea-mask gating, across the angular-tolerance sweep. The trained checkpoint and pipeline scripts are publicly available (see Data Availability Statement).

Algorithm	T = 15°	T = 60°	T = 90°	T = 120°	T = 150°
HOR Horn-Schunck	0.523	0.546	0.568	0.607	0.640
HOR Lucas-Kanade	0.530	0.552	0.566	0.586	0.616
Horn-Schunck (HS)	0.523	0.546	0.569	0.607	0.640
Lucas-Kanade (LK)	**0.593**	0.584	0.609	0.668	**0.686**

On Haulover, the trained six-class segmenter assigns nearly identical mean *P*(sea) inside and outside the rip-current annotation, so a multiplicative sea-gate alone provides little rip-vs-non-rip contrast on this scene. Two further post-processing steps – spatial smoothing and cross-algorithm ensembling, described next – recover Haulover.

### Spatial smoothing and cross-algorithm ensemble

The per-pixel temporal accumulator produces heatmap values that are correct on average but noisy at the pixel scale: each pixel’s count is the result of ~600 strongly temporally-correlated angular tests, each of which is a {0, 1} indicator whose Bernoulli variance dominates at intermediate counts. Rip channels in our two evaluation scenes occupy spatial extents of roughly 50–100 px (across-shore width on the 1920×1080 frame), so a low-pass filter at that scale can be expected to denoise the heatmap without smearing across rip boundaries. We therefore apply a fixed Gaussian blur (σ=25 px, isotropic) to the per-pixel heatmap as a single post-processing step. The per-scene-selected upper bound is insensitive to this choice: varying σ over [15,35] px changes the full-pipeline AUC by less than 0.002 on Palm Beach (0.7052–0.7067) and less than 0.018 on Haulover (0.6886–0.7064); the per-σ values are included in the released result tables.

Independently, the four optical-flow algorithms (HS, HOR HS, LK, HOR LK) make different mistakes – LK fails on textureless regions while HS over-regularizes near sharp boundaries – so a normalized average across algorithms cancels independent errors. Concretely, each algorithm’s heatmap is smoothed with the σ=25 px Gaussian, divided by its own global maximum over the frame, and the resulting unit-scaled maps are averaged pixel-wise.

Tables 6 and 7 report Palm Beach and Haulover AUC under each post-processing variant, at two operating points each: the neutral default $T=90^{\circ }$ and the per-scene optimal tolerance. On Palm Beach, the seg-gated two-algorithm (HOR HS + LK) ensemble with σ=25 smoothing attains 0.636 at the $T=90^{\circ }$ default and reaches 0.7066 at the per-scene optimum $T=150^{\circ }$ (adding HOR LK does not change the result to four-decimal precision; the full *T*-curve of this exact recipe is included in the released result tables). On Haulover, the canonical 2020 accumulators are *natively* computed at the $T=90^{\circ }$ criterion, so the smoothed canonical HOR HS + HOR LK ensemble with sea-mask post-multiplication (0.6599, third row of Table 7) *is* the neutral-default operating point; adding a smoothed seg-gated Lucas-Kanade term at the per-scene-tuned $T=15^{\circ }$ (mixed at 40% weight; like $T=15^{\circ }$, this weight is a per-scene-selected choice, whereas the neutral-default operating point 0.6599 involves no such tuning) lifts this to 0.7023, with the sphere-projected variant of the LK term contributing a small additional improvement to 0.7040 (the Sphere-Projection Contribution subsection).

**Table 6:** Palm Beach AUC contribution per post-processing component, at the angular tolerance where each method peaks. The bottom row is the framework’s full-pipeline result.

Method	*T*	σ	AUC
*Single-algorithm seg-gated, no smoothing*			
HOR Horn–Schunck	150°	–	0.6402
Lucas–Kanade	150°	–	0.6860
*Smoothed + ensembled*			
HOR HS, smoothed	150°	25	0.6514
**HOR HS + LK ensemble, smoothed (neutral default)**	**90°**	**25**	**0.6356**
Same recipe, per-scene optimum (upper bound)	150°	25	0.7066
Same recipe, direction test removed (control)	—	25	0.6183

**Table 7:** Haulover AUC contribution per post-processing component. Each row adds one component to the row above. The canonical 2020 accumulators of the first three rows are natively computed at the $T=90^{\circ }$ criterion, so the third row (0.6599) is the pipeline’s neutral-default operating point; the fourth row adds a seg-gated LK term at the per-scene-tuned $T=15^{\circ }$, and the bottom row adds the sphere projection on top.

Method	AUC
HOR HS canonical, σ=25 smoothed	0.6204
+ HOR LK canonical, σ=25 ensembled (max-normalized)	0.6594
+ Sea-mask post-multiplication	0.6599
+ Smoothed seg-gated LK term, $T\;=\;15^{\circ }$, no projection (w = 0.4)	0.7023
**+ Sphere projection ($\psi \;=\;60^{\circ }$) on the LK term**	**0.7040**

**Source of the canonical Haulover heatmaps.** For full transparency: the HOR HS and HOR LK heatmap arrays used as the canonical base in Table 7 are the output of the original 2020 system on the Haulover scene, retained as fixed intermediate artifacts and will be distributed through the archival data deposit described in the Data Availability Statement rather than through the code repository. The pre-computed dense optical-flow zip that originally fed those heatmaps was not retained and cannot be reconstructed from the raw video alone. The sphere-projected Lucas-Kanade term (last row of Table 7) is, by contrast, a from-scratch re-run on the raw Haulover video, reproducible end-to-end via the scripts in the code repository. The Palm Beach numbers in Table 6 are end-to-end reproducible from the raw Palm Beach video without dependence on any 2020 stored artifact.

Fig 12 shows the resulting full-pipeline heatmaps next to the expert annotations for both scenes. The smoothing and ensemble steps are simple, deterministic post-processing – they require no additional training and cost less than 0.5 s per scene at 1920×1080 resolution.

**Fig 12:**
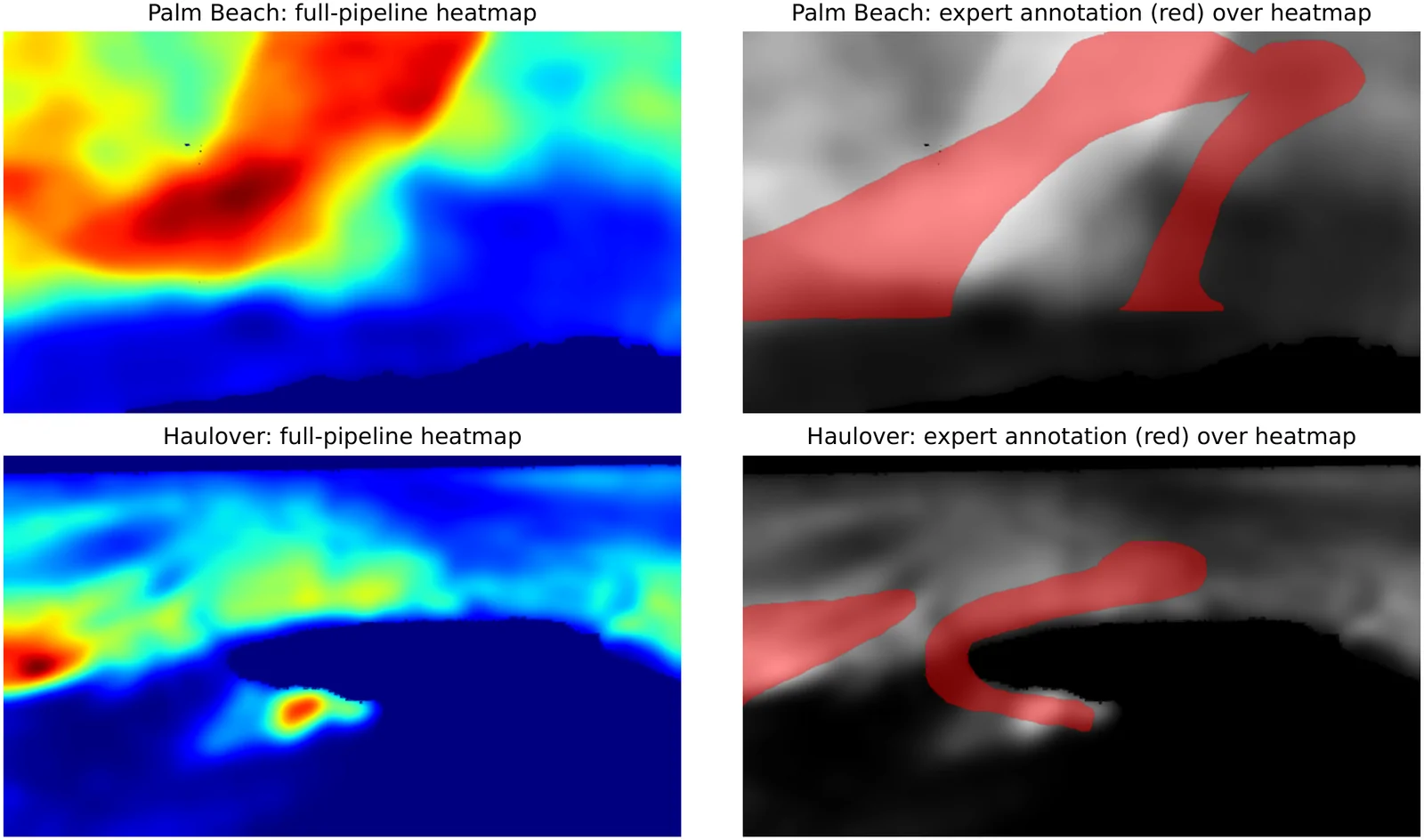
Final full-pipeline output versus expert ground truth. Left column: the full-pipeline rip-current heatmap (Palm Beach top, Haulover bottom) at each scene’s per-scene-selected upper-bound operating point of Table 4. Right column: the same heatmap in grayscale with the three-expert consensus annotation overlaid in red. The highest-scoring regions fall within or adjacent to the annotated rip channels on both scenes, consistent with the PR-AUC values of Table 4.

### Sphere-Projection Contribution

The sphere-projection module of Section ‘Almost Local-Isometric Embedding Modeling on Sphere’ applies a per-pixel Jacobian that maps pixel-space velocity to ground-space velocity. The contribution is identity-equivalent at strict nadir – where the local Jacobian reduces to (h/f)·I, an isotropic scale that preserves angles exactly – and contributes positive AUC only when the camera carries actual non-zero tilt. Palm Beach’s gimballed-nadir capture sits in the former regime, so we ablate the projection on Haulover where the camera carries a measurable forward tilt.

Table 8 reports a three-algorithm, five-tilt sphere sweep on Haulover with the seg-gated pipeline at $T=15^{\circ }$, σ=25 px smoothing, and sea-mask post-multiplication. This configuration isolates the geometric sphere-projection contribution from the smoothing-and-ensembling post-processing block of the Spatial Smoothing and Cross-Algorithm Ensemble subsection: each row reports a single optical-flow algorithm at the strictest angular tolerance, so the absolute AUC values (0.15–0.31) reflect the per-algorithm baseline before ensembling and full-sweep smoothing rather than the framework’s overall detection performance (Haulover full-pipeline AUC: 0.7040, Table 7). The ψ convention follows the manuscript’s spherical-polar parameterization: $\psi =90^{\circ }$ is true nadir; smaller ψ corresponds to greater forward camera tilt. Three findings emerge:

- **Sphere at** $\psi =90^{\circ }$ **reproduces the no-projection AUC exactly** (scaled-identity, angle-preserving Jacobian), confirming the mathematical claim that the projection is a pure pass-through at strict nadir.
- **Forward tilt monotonically lifts AUC on the HS-family** (ψ from $90^{\circ }$ down to $60^{\circ }$). HOR Horn-Schunck rises 0.159→0.200 (+ 0.041, + 26% relative); HOR Lucas-Kanade rises 0.193→0.222 (+ 0.029, + 15% relative). The monotonic improvement across the forward-tilt range – with a declining response at $\psi =100^{\circ }$ (backward tilt) – is consistent with a genuine geometric correction rather than noise. This ablation certifies less than it might appear to: the comparison baseline is *no* projection, and since the planar and sphere models coincide to within a third of a pixel at this altitude (Table 9), a tilt-aware *planar* homography would be expected to reproduce this lift; the measured gain therefore certifies tilt correction generically, not the spherical construction specifically, whose distinct value lies in the high-altitude regime of Table 9.
- **The HS-family best-within-sweep at** $\psi =60^{\circ }$ **corresponds to** $\sim 30^{\circ }$ **forward camera tilt**, broadly consistent with the visual perspective of the Haulover capture; since $60^{\circ }$ is the boundary of the swept range and the response is still rising there, the within-sweep best is a lower bound on the achievable tilt-correction lift rather than a resolved optimum. Plain Lucas-Kanade is closer to its baseline and prefers a slightly smaller tilt ($\psi =80^{\circ }$); LK’s broader spatial support partially absorbs the perspective distortion that the regularized HS variants do not.

**Table 8:** Sphere-projection ablation on Haulover, seg-gated pipeline at $T=15^{\circ }$ with Gaussian smoothing σ=25 px and sea-mask post-multiplication. “no proj” is the same pipeline with the projection skipped. “Lift” = best ψ minus no-projection. Five tilt values cover the manuscript’s spherical-polar ψ range from $60^{\circ }$ ($30^{\circ }$ forward tilt) through $100^{\circ }$ ($10^{\circ }$ backward tilt), with $\psi =90^{\circ }$ as strict nadir.

Algorithm	no proj	$\psi \;=\;60^{\circ }$	$\psi \;=\;70^{\circ }$	$\psi \;=\;80^{\circ }$	$\psi \;=\;90^{\circ }$	$\psi \;=\;100^{\circ }$	Lift
HOR Horn-Schunck	0.159	**0.200**	0.186	0.172	0.159	0.149	+ 0.041
HOR Lucas-Kanade	0.193	**0.222**	0.213	0.203	0.193	0.185	+ 0.029
Lucas-Kanade	0.310	0.312	0.312	**0.313**	0.310	0.305	+ 0.003

**Table 9:** Planar-versus-sphere reconstruction error over the camera’s ground footprint (DJI Phantom-3 optics: *f* = 4 mm, sensor width 6.16 mm; nadir view; ground truth synthesized on the true spherical surface). “Planar RMS/max” is the cost of ignoring Earth curvature, and it tracks the analytic curvature sag of the footprint, which validates the numerics. We also print the sphere model’s own reconstruction residual. Two readings follow. (i) At the 50–70 m study altitude the curvature effect is below one-third of a 1080p pixel, so the planar model is fully adequate — and indeed the sphere residual there (0.05–0.11 cm) *exceeds* the planar error, so this computation cannot resolve the comparison at low altitude; the sphere residual is non-monotone in altitude (0.053→0.112→0.048→0.003 cm), which identifies it as a numerical conditioning floor of the synthetic reconstruction rather than a model error. (ii) Above ~150 m the planar error grows past the sphere residual and reaches decimeters-to-meters in the high-altitude/satellite regime, which is where the general formulation is required.

Altitude	Footprint	Planar RMS	Planar max	Sphere RMS	Curv. sag
50 m	77 m	0.004 cm	0.013 cm	0.053 cm	0.012 cm
70 m	108 m	0.009 cm	0.026 cm	0.112 cm	0.023 cm
150 m	231 m	0.039 cm	0.120 cm	0.048 cm	0.105 cm
500 m	770 m	0.44 cm	1.3 cm	0.003 cm	1.2 cm
1000 m	1540 m	1.8 cm	5.3 cm	0.001 cm	4.7 cm
3000 m	4620 m	16 cm	48 cm	< 0.001 cm	42 cm
10000 m	15400 m	1.8 m	5.3 m	< 0.001 cm	4.7 m

When the sphere-projected Lucas-Kanade term at $\psi =60^{\circ }$ ($T=15^{\circ }$, σ=25; the tilt matching the Haulover capture, used as the ensemble operating point) is added to the canonical-smoothed Haulover ensemble at weight *w*_sphere_ = 0.4, the full-pipeline AUC rises from 0.7023 (no projection) to 0.7040 (with sphere), the + 0.0017 marginal lift reported in Table 7. The smaller *ensemble* contribution relative to the *single-algorithm* HS-family lifts (+ 0.029 to + 0.041) is expected: the ensemble already averages two canonical sources whose tilt-induced angular biases partially cancel, leaving less for the sphere-projected term to recover.

**Two separable effects: tilt correction versus Earth curvature.** The projection module carries out two geometrically distinct operations, which should not be conflated. The first is the *tilt-aware Jacobian*, which corrects the anisotropic velocity distortion introduced by non-nadir camera pose; this is a first-order (perspective) effect that is present at any altitude, and it is the source of the measured + 0.029 to + 0.041 single-algorithm lift on the forward-tilted Haulover capture above. The second is the *Earth-curvature* term, which is a second-order effect that scales with the ground footprint of the frame. Table 9 quantifies the second effect directly: we synthesize a ground grid on the true spherical surface, project it through the camera, and recover it via both a planar tangent-plane homography and the sphere model, reporting the planar model’s error as the cost of ignoring curvature. At the manuscript’s 50–70 m altitude the curvature term costs the planar model at most 0.03 cm — below one-third of a pixel in 1080p video — so it is genuinely negligible for this study. (We do not claim the two models agree to that tolerance: as Table 9 shows, the sphere reconstruction’s own numerical residual is larger than the planar error at these altitudes, so the computation cannot resolve which model is closer to truth below ~150 m. What it does establish is that ignoring curvature costs nothing measurable here.) The same table shows where it ceases to be negligible: the planar RMS error grows to 1.8 cm at 1 km, 16 cm at 3 km, and 1.8 m at 10 km ground altitude, while the peak frame-edge error is approximately 3× larger and matches the analytic curvature sag of the footprint, validating the computation. The planar homography is thus the small-footprint limit of the sphere formulation; the sphere model is retained not for the two UAV scenes here, where the planar model suffices, but as a single expression that remains correct into the high-altitude aerial-survey and satellite regimes.

The full derivation of the almost-local-isometric embedding and its Jacobian is given in the Methods (subsubsection “Almost Local-Isometric Embedding Modeling on Sphere”); this subsection quantifies its contribution and the planar-versus-sphere bound.

### Generalization to additional UAV clips: Short-window evaluation under camera motion

The Haulover capture is close to true hover (phase-correlation cumulative drift ≤23 px over the 20 s window); the Palm Beach capture is not — its cumulative drift reaches ~230 px mid-window before partially returning — so “approximate hover” describes only one of the two primary scenes. Two provenance facts about the sub-clip evaluation should be noted together: the sub-clips come from the same South Beach source captures that supplied the segmenter’s training frames (Table 2), so the seg-gated results on them are *not* scene-independent evidence; at the frame level, however, a dense pixel-MSE search of all 16 sub-clips finds no training frame extracted from any of them (minimum MSE 448, none below 200). To probe whether the framework generalizes beyond these two primary scenes, we ran a frame-aware evaluation on 16 additional sub-clips drawn from the South Beach 1 and South Beach 2 source videos of Table 2 (eight sub-segments per source video — every sub-segment for which an expert annotation exists, with no further curation). For each sub-clip, Dr. Leatherman’s group provided two independent expert annotations: a narrow *rip-outline* annotation (median positive-pixel fraction ≈5%) and a broader *rip-region* annotation (median positive-pixel fraction ≈15%); we evaluate against both. We matched each annotator’s reference image to a specific video frame by phase-correlation on the static portion of the scene; the reference frame indices clustered at ~1,175–1,205 (~20 s into each sub-clip at 60 fps), suggesting a consistent annotation convention.

**Camera motion is not negligible.** A phase-correlation analysis on the first sub-clip of South Beach 1 shows that the UAV is not in strict fixed hover: across the 20-second window the camera drifts at approximately 5–10 px per second on a 1920×1080 frame, with two clearly identifiable pan events at t≈4.5 s and t≈14.5 s. Cumulative drift at the reference frame is 109 px in *x* and −47 px in *y*, modest but enough to blur a per-image-pixel temporal accumulator: pixels at the same image coordinate sample different ground locations across the sub-clip.

**A short stable window matters.** We swept the accumulation window over {5, 10, 15, 20} seconds and three start positions (early, middle, anchored at the reference frame). Results are summarized in Table 10. On the first sub-clip of South Beach 1, the 10-second window anchored at the reference frame attains AUC **0.6276** (HS + LK normalized ensemble, $T=30^{\circ }$, σ=25 px), above the 0.6 pass-threshold we adopt as a per-clip generality bar (a round level chosen for reporting convenience, roughly matching the primary scenes’ neutral-default performance and an order of magnitude above the sub-clips’ 1.6–9.6% chance-level PR-AUC); the corresponding 5-second window reaches 0.6196; and the full 20-second window degrades to 0.5633. The same protocol applied to the remaining 15 South Beach sub-clips and the 2 supplementary YouTube clips lifts the per-sub-clip AUC modestly (median + 0.01) but only this first sub-clip crosses the 0.6 bar. Among the rest, the third sub-clip of South Beach 2 rises from 0.2788 (full) to 0.3657 (late 10 s); the second sub-clip of South Beach 1 rises from 0.4247 to 0.4467. The South Beach sub-clips are a deliberately harder generalization target than Palm Beach or Haulover – smaller rip channels, more variable UAV positioning, and lower rip-to-background visual contrast – and we report the full distribution of peak AUCs (range 0.15–0.63) to document cross-scene performance transparently. Sub-clips with peak AUC in the 0.15–0.32 range indicate that camera motion is one limiting factor but not the only one: clip-to-clip variation in rip visibility, in shoreline geometry, and in the rare-class fraction of the annotation (rip-outline positive rates: 1.6%–9.6% across these sub-clips) jointly bound the achievable AUC.

**Table 10:** Window-sweep AUC summary for the first sub-clip of South Beach 1 (the only sub-clip that crosses the 0.6 pass-threshold under the short-window protocol) together with four representative comparison sub-clips spanning the achievable AUC range. Best entry per row is in bold. “late_5s” / “late_10s” = accumulation window ending at the annotator’s reference frame; “full” = frames 0... ref. All entries are AUC against the narrow rip-outline annotation under the seg-gated pipeline with Gaussian smoothing σ=25 px; the best-performing (algo, *T*) pair per window is reported.

Sub-clip	full (20 s)	late_10s	late_5s	best (algo, *T*)
South Beach 1, segment 1	0.5633	**0.6276**	0.6196	ens(HS + LK), $T\;=\;30^{\circ }$
South Beach 2, segment 3	0.2788	**0.3657**	0.2937	HOR LK, $T\;=\;15^{\circ }$
South Beach 1, segment 2	0.4247	**0.4467**	0.4395	LK, $T\;=\;105^{\circ }$
South Beach 1, segment 5	0.3002	0.3000	**0.3133**	ens(HS + LK), $T\;=\;75^{\circ }$
South Beach 2, segment 7	0.2280	0.2359	**0.2552**	LK, $T\;=\;45^{\circ }$

**Interpretation.** The short-window evaluation on the first sub-clip of South Beach 1 demonstrates that when the UAV’s positional drift is moderate (≲150 px cumulative) and the rip channel is visually distinct from background wave activity, the framework attains AUC ≥0.6 on an additional reported scene under a per-clip stable-window protocol. We do not claim broad cross-scene generalization at this AUC level: of the 18 evaluable sub-clips only this first sub-clip crosses the 0.6 threshold, and the median best AUC across the remaining sub-clips (taking the higher of the two expert annotations per sub-clip) is approximately 0.21. The two limiting factors – UAV positional stability and rare-class precision – jointly motivate the future-work agenda described in the Conclusion: a motion-compensated accumulator that registers each frame to a common reference before voting, and a broader corpus of UAV-perspective rip annotations to disentangle annotator-dependent variance (mean Cohen κ between the rip-outline and the rip-region annotations on these sub-clips is 0.254, indicating substantial disagreement on rip extent across the two independent annotations) from scene-dependent detection difficulty. The per-sub-clip reference frame indices, motion-drift measurements, and per-window AUC table are publicly available (see Data Availability Statement).

### Comparison with a deep-learning baseline

To contextualize the framework against modern learning-based instance segmentation, we trained a YOLOv8m-seg model (Ultralytics, 27.2 M parameters) for 50 epochs on the NTIRE 2026 RipDetSeg challenge dataset [39], which contains 29,307 labeled training images of rip currents drawn from heterogeneous photographic and aerial sources, plus 3,255 held-out validation images. On RipDetSeg’s own validation split, evaluated using the official challenge scoring script, the model achieves F1 at IoU 0.50 of 0.963, F2 at IoU 0.50 of 0.968, F1 averaged over IoU thresholds 0.40–0.95 of 0.803, and a composite NTIRE Score of 0.8855. By external benchmark standards this is a strong baseline.

To probe whether the framework retains a relative advantage over the deep baseline on UAV imagery, we additionally evaluated both methods on six of the South Beach sub-clips of Table 2 (the same source captures that supplied the segmenter’s training frames, though none of the training frames is extracted from these sub-clips). We note the exposure asymmetry explicitly: the YOLOv8 model has zero exposure to these scenes, while our framework’s segmenter was trained on frames from the same captures; the comparison is therefore indicative rather than strictly matched. Each clip carries an independent expert rip-current annotation produced by Dr. Stephen B. Leatherman. We applied the trained YOLOv8m-seg model frame-by-frame (sampled at 2 fps from the 60 fps source) and aggregated per-pixel maximum confidence across the clip. We then compared the resulting rip-likelihood map against the framework’s HOR Horn-Schunck heatmap on the same clip, evaluated against the same expert annotation. Because the absolute precision-recall AUC values on these clips are sensitive to the rare-class fraction in each annotation (typically 1–10% rip pixels), we do not report them as performance numbers; instead, we use them only to compare the two methods on identical evaluation footing.

The framework’s HOR Horn-Schunck heatmap exceeded the YOLOv8m-seg AUC on 4 of the 6 clips. The two clips on which YOLOv8 won are visually closer to RipDetSeg’s training distribution (clear rip channel with photographic-style framing), while the framework wins on clips with weaker visual contrast but coherent offshore drift – a regime where temporal motion priors recover signal that a per-frame visual classifier misses. Notably, on the strongest-signal clip among the six, YOLOv8 detected nothing in 21 sampled frames (mean confidence < 0.01) despite having seen 29,307 rip-current images during training, while the framework produced a clear rip-channel heatmap. We read this comparison not as a sweeping claim that classical optical flow beats deep learning – the deep model would likely exceed the framework if fine-tuned on a sufficiently large UAV-perspective rip annotation corpus, which we do not have – but rather as evidence that the framework’s domain-specific physical priors (offshore direction, temporal accumulation) recover useful signal in regimes where a general-purpose visual detector trained on out-of-distribution data fails. We view the two as complementary, and an ensemble of the optical-flow framework and a UAV-fine-tuned deep detector is a natural direction for future work.

### Computational performance

We report per-stage runtime on two reference platforms: an Intel-based 16-core CPU workstation (no GPU acceleration in the optical-flow stage) and an embedded UAV-deployable NVIDIA Jetson TX2 module. Per-frame timings on the workstation are approximately 0.45 s for HOR Horn-Schunck dense optical flow at 1920×1080 resolution (single thread), or ~0.04 s per frame pair on the same hardware with 12-worker process-pool parallelism. The offshore-direction estimation runs in ~1 s per scene on commodity hardware (using cv2.distanceTransform for the distance-to-coast field; the original implementation iterated in Python and was substantially slower). The temporal heatmap accumulation runs in < 0.1 s per frame at 1920×1080 resolution. End-to-end, processing a 20 s clip (600 frame pairs at 30 fps) takes approximately 4 minutes on the 16-core workstation with no GPU acceleration – slower than real-time on this platform, but the workload parallelizes trivially across frame pairs and would benefit substantially from GPU port (the wavelet decomposition and Horn-Schunck iterations are the dominant cost). On the Jetson TX2, the same 20 s clip processed to a heatmap in approximately 45 s under the original 2020 measurement; the TX2 is more constrained than current Jetson Orin parts, and an updated embedded measurement is left to future work.

## Conclusion and further work

This paper proposes what is, to our knowledge, the first end-to-end pipeline that detects rip currents at the pixel level from raw UAV video without manual ground-control points. The pipeline combines a CNN-based six-class scene parser, a sphere-aware projection from image to ground coordinates with a tilt-aware Jacobian correction, dense optical-flow estimation with five algorithm variants, a per-pixel temporal accumulator that flags pixels whose flow direction agrees with the locally-estimated offshore direction within the neutral tolerance $T=90^{\circ }$ (the offshore half-plane), and a post-processing block of Gaussian smoothing (σ=25 px) plus normalized cross-algorithm ensembling, with the CNN sea-gate and sphere-projection modules engaged per scene where they contribute (the sea-gate on Palm Beach, the sphere-projected term on Haulover; Tables 6 and 7). We evaluated the framework on two UAV-captured rip-current scenes (Palm Beach and Haulover Park, Florida) with three-expert consensus annotations. At the physically-neutral $T=90^{\circ }$ default the framework attains precision-recall AUC of 0.636 at Palm Beach and 0.660 at Haulover; per-scene selection — of the angular tolerance, the ensemble membership, the Haulover mixing weight *w* = 0.4, the sphere tilt $\psi =60^{\circ }$, and which modules are engaged on which scene — against the same expert annotation used for evaluation raises these to 0.7066 and 0.7040, which we report as an optimistic upper bound rather than an expected-performance estimate. We report the default as the headline because the tuned values are selected on the evaluation data itself, and we quantify what the direction test alone contributes with a control in which it is removed entirely (0.618 on Palm Beach). We additionally trained and released a YOLOv8m-seg deep-learning baseline on the NTIRE 2026 RipDetSeg challenge corpus (29,307 labeled images, composite NTIRE Score 0.8855), which we view as complementary to rather than directly competitive with the optical-flow framework given the substantial domain gap between the heterogeneous web-scraped training set and the manuscript’s specific UAV viewing geometry.

The current study has several limitations that we note explicitly to scope the contribution and motivate future work. First, the framework assumes an approximately straight shoreline with uniform offshore bathymetry; embayed beaches, headland-controlled rips, and complex jetty deflection (such as the lower-middle portion of the Haulover scene) violate this assumption and the framework produces low AUC there. Second, optical flow measures the apparent motion of visible surface tracers, not the water velocity directly; the method is therefore valid only where breaking-wave foam, fluorescein dye, or sediment fronts act as tracers advected by the surface current, and it degrades under specular glare, glassy (tracer-free) water, or fully submerged flow [8,12]. The direction-consistent temporal accumulation formalized in the Methods suppresses the zero-mean wave and glint components (with a signal-to-noise gain $\propto \sqrt{N}$; Fig 11), but it cannot recover a rip whose surface signature is absent, and classical dense optical-flow methods remain sensitive to lighting, foam aliasing, and turbulent eddies that the accumulation step compensates partially but not fully. Third, evaluations are conducted in favorable weather; the framework has not been tested under rain, fog, or wind speeds above ~8 m/s. Fourth, our virtual-drifter visualization (Fig 5) is internal to the same velocity field that drives detection, so it should be read as a consistency check rather than an independent validation; future work should obtain GPS-drifter or fluorescein-dye reference tracks for an independent validation. Fifth, our quantitative evaluation is restricted to two scenes for which expert consensus annotations were available — both on the southeast Florida Atlantic coast — so geographic and morphological diversity is limited, and broader evaluation across more diverse coastal geometries is the natural next step; in the 18-sub-clip cross-scene test only one sub-clip exceeds AUC 0.6 (median best ≈0.21), so demonstrated generalization beyond the two primary scenes is currently limited. Sixth, ground truth for the sub-clip analysis carries appreciable annotator subjectivity (mean Cohen κ=0.254 between the two independent annotations), and the accumulator assumes a quasi-stationary camera: UAV positional drift beyond ~150 px cumulative degrades the temporal vote. Finally, all captures are daylight recordings, and the current CPU implementation runs slower than real time, so in-field real-time deployment remains to be demonstrated.

Future work has three priorities. (1) Expand evaluation to a more diverse coastal corpus that covers rocky coasts, varying beach geomorphologies, sediment-laden water, low-light and high-wind conditions; collect labeled UAV-perspective rip-current annotations sufficient to fine-tune a deep detector for a fair head-to-head comparison. (2) Replace the heuristic HSV foam-color threshold with a learned segmentor; the YOLOv8m-seg model trained in this work could serve as a drop-in replacement for the wave-mask step once UAV-perspective rip annotations are available. (3) Move the optical-flow stage to GPU and benchmark on a UAV-deployable platform (e.g., NVIDIA Jetson Orin) for in-field deployment; we report CPU-only timing in this paper as the platform context the original 2020 measurements assumed.

## References

[1] US Department of Commerce, National Oceanic and Atmospheric Administration. Surf Zone Fatalities in 2019: 90. 2020. Available from: https://www.weather.gov/safety/ripcurrent-fatalities

[2] Branche CM, Stewart S. Lifeguard effectiveness: a report of the working group. National Center for Injury Prevention and Control, Centers for Disease Control and Prevention; 2001.

[3] Haller MC, Dalrymple RA. Rip current instabilities. J Fluid Mech. 2001;433:161–92. doi: 10.1017/s0022112000003414

[4] Haller MC, Honegger D, Catalan PA. Rip Current Observations via Marine Radar. J Waterway Port Coastal Ocean Eng. 2014;140(2):115–24. doi: 10.1061/(asce)ww.1943-5460.0000229

[5] Kumar N, Voulgaris G, Warner JC. Implementation and modification of a three-dimensional radiation stress formulation for surf zone and rip-current applications. Coast Eng. 2011;58(12):1097–117. doi: 10.1016/j.coastaleng.2011.06.009

[6] Brown J, MacMahan J, Reniers A, Thornton E. Surf zone diffusivity on a rip‐channeled beach. J Geophys Res. 2009;114(C11). doi: 10.1029/2008jc005158

[7] Wang H, Zhu S, Li X, Zhang W, Nie Y. Numerical simulations of rip currents off arc-shaped coastlines. Acta Oceanol Sin. 2018;37(3):21–30. doi: 10.1007/s13131-018-1197-1

[8] Holman R, Haller MC. Remote sensing of the nearshore. Ann Rev Mar Sci. 2013;5:95–113. doi: 10.1146/annurev-marine-121211-172408 22809186

[9] Chickadel CC, Holman RA, Freilich MH. An optical technique for the measurement of longshore currents. J Geophys Res. 2003;108(C11). doi: 10.1029/2003jc001774

[10] Holland KT, Puleo JA, Kooney TN. Quantification of swash flows using video-based particle image velocimetry. Coast Eng. 2001;44(2):65–77. doi: 10.1016/s0378-3839(01)00022-9

[11] Kennedy AB, Thomas D. Drifter measurements in a laboratory rip current. J Geophys Res. 2004;109(C8). doi: 10.1029/2003jc001927

[12] Derian P, Almar R. Wavelet-Based Optical Flow Estimation of Instant Surface Currents From Shore-Based and UAV Videos. IEEE Trans Geosci Remote Sens. 2017;55(10):5790–7. doi: 10.1109/tgrs.2017.2714202

[13] Sun A, Tao Y, Shyu ML, Chen SC, Blizzard A, Rothenberg WA, et al. Multimodal data integration for interactive and realistic avatar simulation in augmented reality. In: 2021 IEEE 22nd International Conference on Information Reuse and Integration for Data Science (IRI). IEEE; 2021. p. 362–9.

[14] Gallop SL, Bryan KR, Coco G. Video observations of rip currents on an embayed beach. J Coast Res. 2009;49–53.

[15] Streser M, Carrasco R, Horstmann J. Video-Based Estimation of Surface Currents Using a Low-Cost Quadcopter. IEEE Geosci Remote Sens Lett. 2017;14(11):2027–31. doi: 10.1109/lgrs.2017.2749120

[16] Sonu CJ. Field observation of nearshore circulation and meandering currents. J Geophys Res. 1972;77(18):3232–47. doi: 10.1029/jc077i018p03232

[17] Inman DL, Zampol JA, White TE, Hanes DM, Waldorf WB, Kastens KA. Field measurements of sand motion in the surf zone. In: Proceedings of the 17th International Conference on Coastal Engineering. American Society of Civil Engineers. 1980. p. 1215–34.

[18] Brander RW, Short AD. Morphodynamics of a large-scale rip current system at Muriwai Beach, New Zealand. Mar Geol. 2000;165(1–4):27–39. doi: 10.1016/s0025-3227(00)00004-9

[19] Short AD, Hogan CL. Rip currents and beach hazards: their impact on public safety and implications for coastal management. J Coast Res. 1994;197–209.

[20] Schmidt WE, Woodward BT, Millikan KS, Guza RT, Raubenheimer B, Elgar S. A GPS-Tracked Surf Zone Drifter*. J Atmos Ocean Technol. 2003;20(7):1069–75. doi: 10.1175/1460.1

[21] Sabet BS, Barani GA. Design of small GPS drifters for current measurements in the coastal zone. Ocean Coast Manage. 2011;54(2):158–63. doi: 10.1016/j.ocecoaman.2010.10.029

[22] Smith JA, Largier JL. Observations of nearshore circulation: Rip currents. J Geophys Res. 1995;100(C6):10967–75. doi: 10.1029/95jc00751

[23] Lushine JB. A study of rip current drownings and related weather factors. Natl Weather Dig. 1991;16:13–9.

[24] Lascody RL. East central Florida rip current program. Natl Weather Dig. 1998;22(2):25–30.

[25] Holman RA, Stanley J. The history and technical capabilities of Argus. Coast Eng. 2007;54(6–7):477–91. doi: 10.1016/j.coastaleng.2007.01.003

[26] Bae JS, Yoon SB, Choi J. Boussinesq modelling of a rip current at Haeundae Beach in South Korea. J Coast Res. 2013;65:654–9. doi: 10.2112/si65-111.1

[27] Yoon SB, Park WK, Choi J. Observation of Rip Current Velocity at an Accidental Event by using Video Image Analysis. J Coast Res. 2014;72:16–21. doi: 10.2112/si72-004.1

[28] Horn BKP, Schunck BG. Determining optical flow. In: Techniques and Applications of Image Understanding. vol. 281. International Society for Optics and Photonics; 1981. p. 319–31.

[29] Lucas BD, Kanade T. An Iterative Image Registration Technique with an Application to Stereo Vision. In: Proceedings of the 7th International Joint Conference on Artificial Intelligence, IJCAI ’81, Vancouver, BC, Canada, August 24-28, 1981. 1981. p. 674–9.

[30] Kadri-Harouna S, Dérian P, Héas P, Mémin E. Divergence-Free Wavelets and High Order Regularization. Int J Comput Vis. 2012;103(1):80–99. doi: 10.1007/s11263-012-0595-7

[31] Dérian P, Mauzey CF, Mayor SD. Wavelet-Based Optical Flow for Two-Component Wind Field Estimation from Single Aerosol Lidar Data. J Atmos Ocean Technol. 2015;32(10):1759–78. doi: 10.1175/jtech-d-15-0010.1

[32] Teed Z, Deng J. RAFT: Recurrent All-Pairs Field Transforms for Optical Flow. In: European Conference on Computer Vision (ECCV). 2020. p. 402–19.

[33] Maryan C, Hoque MT, Michael C, Ioup E, Abdelguerfi M. Machine learning applications in detecting rip channels from images. Appl Soft Comput. 2019;78:84–93. doi: 10.1016/j.asoc.2019.02.017

[34] de Silva A, Mori I, Dusek G, Davis J, Pang A. Automated rip current detection with region based convolutional neural networks. Coast Eng. 2021;166:103859. doi: 10.1016/j.coastaleng.2021.103859

[35] Zhu D, Qi R, Hu P, Su Q, Qin X, Li Z. YOLO-Rip: A modified lightweight network for Rip currents detection. Front Mar Sci. 2022;9. doi: 10.3389/fmars.2022.930478

[36] Dumitriu A, Tatui F, Miron F, Ionescu RT, Timofte R. Rip current segmentation: A novel benchmark and YOLOv8 baseline results. In: Proceedings of the IEEE/CVF Conference on Computer Vision and Pattern Recognition (CVPR) Workshops. 2023. p. 1261–71.

[37] Dumitriu A, Tatui F, Miron F, Ralhan A, Ionescu RT, Timofte R. RipVIS: Rip currents video instance segmentation benchmark for beach monitoring and safety. In: Proceedings of the IEEE/CVF Conference on Computer Vision and Pattern Recognition (CVPR). 2025. p. 3427–37.

[38] de Silva A, Zhao M, Stewart D, Khan FH, Dusek G, Davis J, et al. RipViz: Finding Rip Currents by Learning Pathline Behavior. IEEE Trans Vis Comput Graph. 2024;30(7):3930–44. doi: 10.1109/TVCG.2023.3243834 37022897

[39] NTIRE 2026 Rip Current Detection and Segmentation Challenge. RipDetSeg: Rip Current Detection and Segmentation Challenge Dataset (v1.1.6); 2026. CVPR 2026 NTIRE Workshop. Available via Codabench Competition 12730. Available from: https://www.codabench.org/competitions/12730/

[40] Ronneberger O, Fischer P, Brox T. U-Net: Convolutional Networks for Biomedical Image Segmentation. In: Navab N, Hornegger J, Wells WM III, Frangi AF, editors. Medical Image Computing and Computer-Assisted Intervention. vol. 9351 of Lecture Notes in Computer Science. Springer; 2015. p. 234–41.

[41] He K, Zhang X, Ren S, Sun J. Deep Residual Learning for Image Recognition. In: IEEE Conference on Computer Vision and Pattern Recognition. IEEE Computer Society; 2016. p. 770–8.

[42] Castelle B, Scott T, Brander RW, McCarroll RJ. Rip current types, circulation and hazard. Earth-Sci Rev. 2016;163:1–21. doi: 10.1016/j.earscirev.2016.09.008

[43] MacMahan JH, Thornton EB, Reniers AJHM. Rip current review. Coast Eng. 2006;53(2–3):191–208. doi: 10.1016/j.coastaleng.2005.10.009

[44] Leatherman SB. Rip Current Measurements at Three South Florida Beaches. J Coast Res. 2017;335:1228–34. doi: 10.2112/jcoastres-d-16-00124.1

[45] Leatherman SB, Leatherman SP. Techniques for Detecting and Measuring Rip Currents. Int J Earth Sci Geophys. 2017;3(1). doi: 10.35840/2631-5033/1814

[46] Sun A. UAV-Video-Based Rip Current Detection in Near-Shore Areas [Master’s thesis]. Miami (FL): College of Engineering, University of Miami; 2020.

